# Crowdsourcing the Measurement of Interstate Conflict

**DOI:** 10.1371/journal.pone.0156527

**Published:** 2016-06-16

**Authors:** Vito D’Orazio, Michael Kenwick, Matthew Lane, Glenn Palmer, David Reitter

**Affiliations:** 1 School of Economic, Political, and Policy Sciences, University of Texas at Dallas, Richardson, TX, United States of America; 2 Department of Political Science, The Pennsylvania State University, University Park, PA, United States of America; 3 College of Information Sciences and Technology, The Pennsylvania State University, University Park, PA, United States of America; Qom University, IRAN, ISLAMIC REPUBLIC OF

## Abstract

Much of the data used to measure conflict is extracted from news reports. This is typically accomplished using either expert coders to quantify the relevant information or machine coders to automatically extract data from documents. Although expert coding is costly, it produces quality data. Machine coding is fast and inexpensive, but the data are noisy. To diminish the severity of this tradeoff, we introduce a method for analyzing news documents that uses crowdsourcing, supplemented with computational approaches. The new method is tested on documents about Militarized Interstate Disputes, and its accuracy ranges between about 68 and 76 percent. This is shown to be a considerable improvement over automated coding, and to cost less and be much faster than expert coding.

## Introduction

The study of political conflict relies heavily on data generated from the content analysis of news reports. Thus far, there have been two primary methods for conducting such analyses: using highly-trained, expert coders or using machines and automated processes. Both experts and machines have benefits and drawbacks for collecting conflict data. While expert coders are expensive and slow, they are capable of accurate, valid data collection. Alternatively, machine coding can quickly classify vast quantities of information at very low cost. The data produced using these automated methods, however, tend to be noisy, limiting the ability to accurately measure concepts of interest. As a result, researchers are faced with the dilemma of either sacrificing cost for quality or sacrificing the quality of data for a reduction in time and cost.

Crowdsourcing has emerged as an alternative to standard methods of data collection. In general, crowdsourcing refers to the act of outsourcing a task “to an undefined (and generally large) network of people in the form of an open call” [1]. In our application, crowdsourcing refers to a process in which a large number of non-experts are asked a series of questions as a means of gathering information on conflict events from news sources. These non-experts are recruited through Amazon’s Mechanical Turk. Like machines, crowdsourcing is fast, cheap, and capable of collecting data on a large scale. Like experts, crowdsourcing utilizes human intelligence to summarize documents and understand the context in which events are taking place.

While crowdsourcing has proven effective in many tasks that were previously believed to require expert knowledge [2–4], it has not been applied to large-scale measurement or data collection tasks in the field of conflict studies. Given the large number of data projects that exist in this field of research, and the even larger number of data projects that *could* exist if not for the costs of expert data collection, an accurate and efficient method of data collection using crowdsourcing could prove particularly valuable.

We propose and assess a hybrid method for coding conflict data that supplements crowdsourcing with computational approaches in an effort to produce accurate data efficiently (henceforth referred to as the crowdsourcing method). Crowdsourcing is compared against machine and expert coding with respect to the ability to measure militarized interstate incidents (MIIs) [5, 6]. These methods are assessed based on accuracy, financial cost, and time to completion. We choose the MII as our construct of interest because the data collection procedures for MIIs are widely known and can be replicated with reasonable accuracy. More importantly, MIIs are representative of many theoretical constructs in conflict research, and political science research more broadly, in that they are complex social processes that are classified with a defined set of coding rules and primarily measured using news reports.

We begin our analysis by using existing methods of expert coding and machine classification. First, expert coders read a set of 450 news documents to identify whether each document contains information on a militarized dispute and, if so, which state was responsible for initiating hostilities, which state was the target, and which specific type of militarized action took place, according to the MII coding rules. Each document is then coded using Tabari, open-source software for generating event data from text documents. The use of expert coding provides us with an inter-coder reliability benchmark and a gold standard classification for each document, as well as an estimated cost for obtaining this coding. The use of Tabari provides us with a benchmark (in addition to expert coding) against which the accuracy of crowdsourcing can be assessed. We then outline the crowdsourcing infrastructure developed for this study.

The crowdsourcing method uses worker recruitment services to obtain between six and ten workers to classify each news story. We provide these workers with a questionnaire designed to obtain information that can be used to identify which documents contain information about MIIs, as well as the relevant features of those disputes. Building on this basic infrastructure, we further combine standard crowdsourcing techniques with computational methods, specifically named entity recognition and Bayesian network aggregation, to improve the quality of the data collected. The results indicate that crowdsourcing is an increasingly viable option for data collection, as the crowd identifies the pertinent aspects of a news document with significantly greater accuracy than machine coding. Although the crowdsourcing method is cheaper than expert coding in terms of monetary cost, the greater advantage is in time to completion. For expert coding, time frames are weeks and months; for crowdsourcing, they are hours and days.

Our contributions are in the application and extension of crowdsourcing techniques to the content analysis of news reports about international conflict. Consistent with research on other topics assumed to required expert coders [2–4]), we find that the crowd in aggregate provides satisfactory results. We also find that the crowd outperforms the fully automated approach.

We extend existing methods for improving crowdsourced judgments to the domain of conflict data collection. Generally, the benefits of supplementing human knowledge with computationally generated information to improve human judgements [7, 8], and the “wisdom of the crowd” [9–11], are well known. In our application, we include named entity recognition for political actors and a Bayesian network classifier to improve the accuracy of the individual crowd responses and the aggregate predictions, respectively.

## Crowdsourcing and Data Collection

Measuring social concepts is a fundamental task in the social sciences [12–15]. Traditionally, this has been a resource-intensive undertaking in which scholars and their research assistants search and read a vast quantity of documents toward locating events of interest that satisfy their operational definition. Some of the largest projects in political science have devoted immense resources to this task, including the Polity and Correlates of War projects. Certainly, these resources have been put to good use. Nevertheless, given recent technological advances and the increasing prevalence of real-time, big data analyses, these traditional approaches to data collection are becoming increasingly antiquated.

Machine coding is a more recent, alternative method for analyzing the content of news reports [16–18]. While machine coding is capable of producing data in near-real-time at marginally zero cost [19], the resulting data have yet to be shown to accurately measure many concepts of interest. Such “noisy” output, or data with a large amount of measurement error, is a consequence of construction; machine coded data commonly map subject and predicate phrases in a news document to a dictionary of terms that are associated with a particular concept [20]. This process codes events in isolation from other events and without any knowledge of the broader political atmosphere.

Crowdsourcing, which uses distributed labor from a community of individuals, is an alternative approach that addresses the primary drawbacks of the expert and machine coded methods. Crowdsourcing has been widely used to find subjects for experimental studies in the behavioral and social sciences [21–23]. Platforms such as Amazon’s Mechanical Turk (AMT) have been evaluated for precisely this task [24]. In our application, we are not recruiting subjects for experimentation, but rather are recruiting non-expert workers to perform classification tasks.

Some of the more common uses of crowdsourcing for classification tasks are in the field of natural language processing [25–28]. Studies such as these have shown crowdsourcing to be an effective tool for solving problems that were previously thought to require substantive expertise. Most closely related to our application, [2] and [29] have used crowdsourcing to measure political ideology and regime types, respectively. Each of these studies were successful in using crowdsourcing to provide quality data efficiently.

These findings suggest that crowdsourcing is likely to be cheaper and faster than expert coding and more accurate than machine coding. However, few, if any, studies have explored the potential to use crowdsourcing to accurately code targeted events from observational reports, such as news documents. As a result, we do not know if crowdsourcing is cheap enough, fast enough, or accurate enough for data collection projects in conflict research, which commonly relies on data coded from such sources. This study quantifies these comparisons for coding MIIs, a commonly used measure of international conflict.

### Coding the Militarized Interstate Incident

Militarized Interstate Disputes (MIDs) are “united historical cases of conflict in which the threat, display or use of military force short of war by one member state is explicitly directed towards the government, official representatives, official forces, property, or territory of another state” [6]. Each MID is comprised of at least one and potentially hundreds of Militarized Interstate Incidents (MIIs). Each MII “is defined as a single military action involving an explicit threat, display, or use of force by one system member state towards another system member state” [6], and are frequently described in a single news report. The traditional method for collecting data on MIIs, as undertaken by the MID3 and MID4 projects [5, 30], is to query LexisNexis and retrieve a document set, manually code the retrieved set with the assistance of highly-trained research assistants, and then for lead researchers to verify the coding—a process common to many subfields.

As our first step, expert coders from the MID4 project–three researchers responsible for collecting, coding, validating, and publishing the MID4 data–replicated this process by individually coding 450 documents of MII-related news stories. To obtain this sample, we drew upon documents identified by the MID4 project as potentially containing information about MIIs. This set of potentially relevant documents was gathered by querying LexisNexis using terms related to militarized incidents and then classifying the documents using support vector machines to obtain the subset of stories most likely to be related to MIIs [31]. We then grouped these stories by year (2007, 2009, and 2010), and manually identified those that likely contained information on MIIs and those that likely did not. This is the same collection and separation process used in the MID4 project, with the same set of documents used to construct the original MID4 data. Further description of the original MID4 collection and coding process can be found in [5]. For each year, we then randomly sampled 100 stories from the positive bin and 50 stories from the negative bin, providing a total of 450 documents for coding. Next, three experts in MII data collection independently coded each of the 450 news documents, identifying whether a MII took place and if so the actors and action involved.

#### The “Gold Standard”

Having established a set of codings for each news document, the experts generated a “gold standard” classification for assessing the accuracy of the crowdsourcing and machine coding methods. To do so, the classifications of each document were examined. When the three experts agreed on a decision, this was accepted as the “correct” classification. When experts disagreed, they were convened and mutually reached agreement after their individual coding. A similar process was used in the original coding of the MID4 data [5]. The “gold standard” is assumed to be correct.

Note that, in creating the gold standard, experts did not employ the same questionnaire architecture used by the crowd, nor did the experts code events in the way Tabari, the automated event coder, codes events. Instead, our method represents simple consensus building, which is often used in data collection projects involving small groups of expert coders.

This approach was taken because our intent was to assess these three methods in coding MIIs from a single document set. We did not evaluate expert coders against crowd workers in their ability to answer the questionnaire correctly, nor did we evaluate expert coders against automated methods to code event data. Our design creates a potentially confounding variable: the *quality* of both the questionnaire and the automated coder. While future research should explore the ways in which the quality of the questionnaire and automated coder can be improved, this was not our primary intent. Rather, our intent was to assess these three methods as they are most commonly used in the construction of conflict data.

#### Inter-Coder Agreement

A common measure of intercoder reliability allows us to assess the level of homogeneity between coders’ classifications of events [32, 33]. The experts’ codings focused on the three most central aspects of MII classification: the initiator state, the target state, and the primary militarized action of the event. We used Fleiss’ Kappa to calculate the intercoder reliability. This measures the direct agreement between coders while accounting for agreement that could occur by chance, was used to calculate the intercoder reliability for each of these fields [33–36]. Kappa coefficients range from zero to one, with measures between 0.61 and 0.8 generally considered “good” levels of intercoder agreement and coefficients between 0.81 and 1.00 being generally considered “very good” or nearly perfect agreement [33].

Table 1 provides the Kappa coefficients between the three expert coders for the three MII attributes of interest. Based on the conventional intercoder reliability scaling, there is “good” or substantial agreement between the expert coders for all three characteristics.

**Table 1:** Intercoder Reliability.

MII Classification	Kappa Coefficient
Initiator	0.7786
Target	0.7643
Militarized Action	0.7025

**Note:** Kappa coefficients of 1.00 reveal perfect agreement between all coders. Conversely, Kappa coefficients of 0.0 mean levels of agreement perfectly predicted by chance.

This agreement notwithstanding, there was also clear heterogeneity between their classifications of the news documents. Even when provided with uniform coding rules, experts’ subjective judgments can lead to differences in the final data produced. Though expert coders may generally produce highly accurate data classifications, there is clearly variation in the interpretation and application of the coding rules. This also underscores the notion that the MII is a complex social construct and is difficult to code consistently.

#### Distribution of incidents by action type

**Fig 1:**
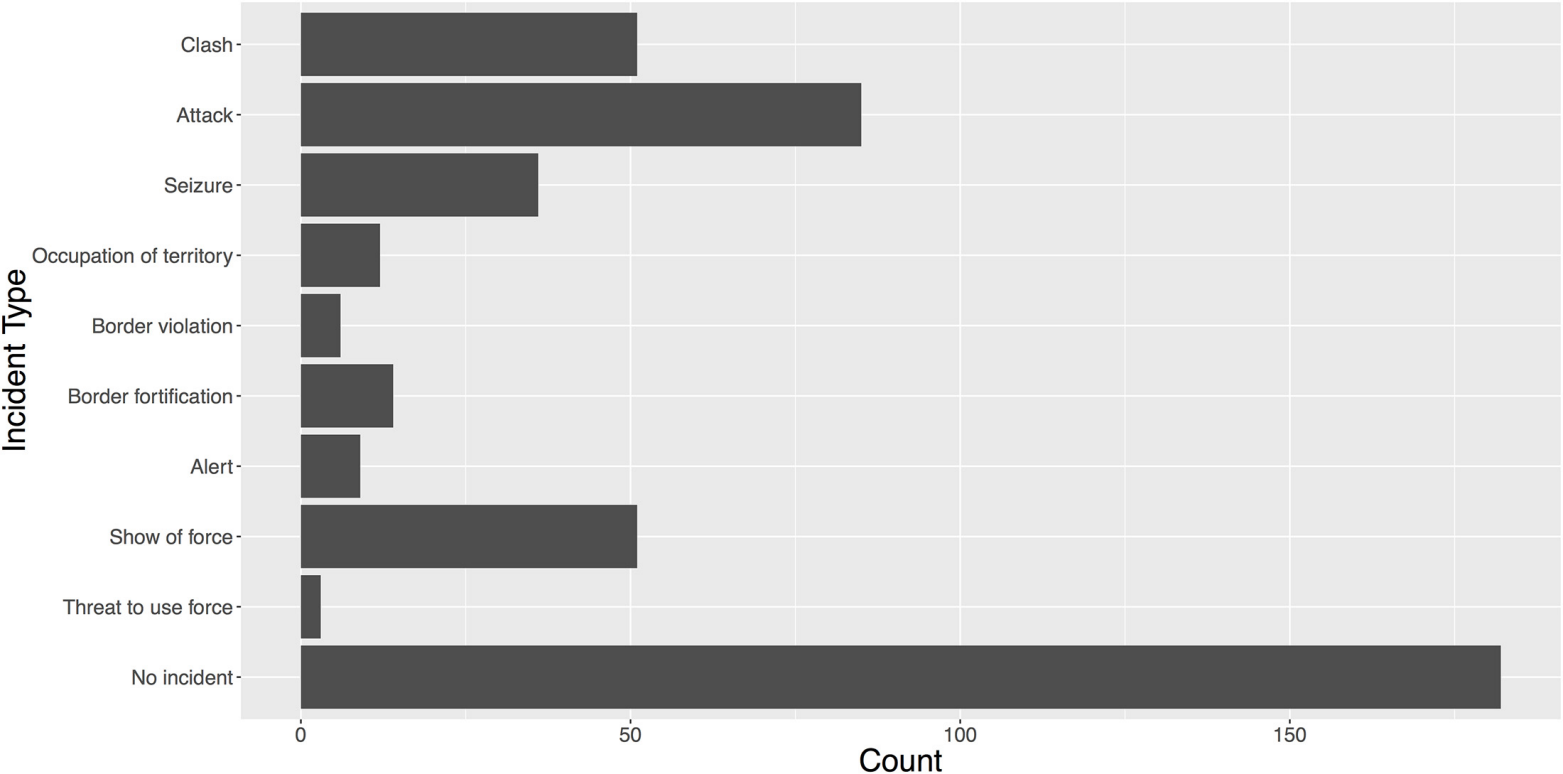
Incident Distribution in Documents. Figure reports the frequency of news documents pertaining to each MII action type selected to develop the gold standard.

As previously stated, each news story is coded to determine which type of militarized action (if any) took place, and if so, which states were involved. Though these actions can be broadly categorized as threats, displays, or uses of force, the expert classifications identifies more specific action types according to the MII classification rules. The distribution of these action types according to the gold standard, is shown in Fig 1. The majority of news stories do not contain any information about MIIs. The next most common category pertains to uses of force, which includes actions like: attacks (where the armed forces of one state fire upon the population or territory of another); clashes (where the armed forces of two states engage in sustained hostilities); seizures (where one state’s armed forces capture another state’s personnel or goods); and occupation of territory (where the armed forces from one state occupies territory belonging to another). Shows of force are the second most common type of MII and include actions such as: border violations (the crossing of a territorial land boundary); border fortifications (an explicit, non-routine demonstration of control over a border area); alerts (an increase in the readiness of a state’s armed forces); and shows of force (a public demonstration of a states military forces intended to intimidate another state). Finally threats to use force, where one state makes a militarized threat conditional on the action of another, are the least common type of MII.

Critically, the distribution of news stories according to MII type displayed in Fig 1 is generally consistent with the known distribution of news stories that the MID4 project origionally used to classify MIIs [5]. This provides a degree of confidence that the reported accuracies of the automated and crowdsourcing methods are a fair estimate of the expected accuracies that would be obtained if the larger population of news stories used for MII data collection.

## Analysis of Automated Machine Classification

Machine coding is an alternative to expert coding that is scalable, efficient, and can be virtually free of cost [17, 18, 37]. Despite these advantages, we find that this method is not accurate in this application, with accuracy levels for actor and action classification ranging between about 30 to 45 percent.

To machine code the documents, we used Tabari [38] and the Conflict and Mediation Event Observations (Cameo) ontology [39]. Specifically, we used Tabari version TABARI.0.8.4b2 and Cameo verbs version CAMEO.091003.master.verbs. The actors files used are: nouns_adj_null.110124.txt, Phoenix.Countries.140130.actors.txt, Phoenix.Internatnl.140130.actors.txt, and Phoenix.MNSA.140131.actors.txt. The agent file is Phoenix.140127.agents.txt, and the options file is the default pipeline.options.txt. All software has been retrieved from https://github.com/openeventdata, and is available there.

Tabari produces event data that detail which actor initiated an action, which actor was the target, the CAMEO action type, and the date on which the event occurred. To discern the codeable MII attributes from this dataset, each CAMEO action type was mapped to a MID code using a deterministic set of rules. For each document, the machine coded MII attributes–initiator, target, action–were compared to the expert coded MII attributes.

Note that there are a small number of MII action types for which there is no corresponding Cameo action code. As a result Tabari is unable to achieve 100 percent accuracy in classifying MIIs. Specifically, in our document set Tabari is unable to correctly classify the 20 news stories containing information on border fortifications and border violations. Because we are interested in Tabariś ability to code MIIs specifically and not political conflict more broadly, we do not drop these cases when computing accuracy. Doing so would not, however dramatically alter our findings or alter the substantive conclusions we draw below.

Of the 450 news stories, Tabari was only able to identify actors and actions in 275. The remaining 175 documents received a *null* coding. A *null* can be produced if no CAMEO event occurred, if the document lacked the specific verbiage required for Tabari to recognize the CAMEO event, or because of technical issues such as a failure to correctly parse complex sentence structures. 91 stories were identified as non-MIIs according to the Gold Standard, so the null result roughly corresponds with reality. The remaining 84 stories were classified as various types of MIIs in the Gold Standard, suggesting Tabari is incapable of recognizing or processing a substantial number of MII relevant stories.

**Fig 2:**
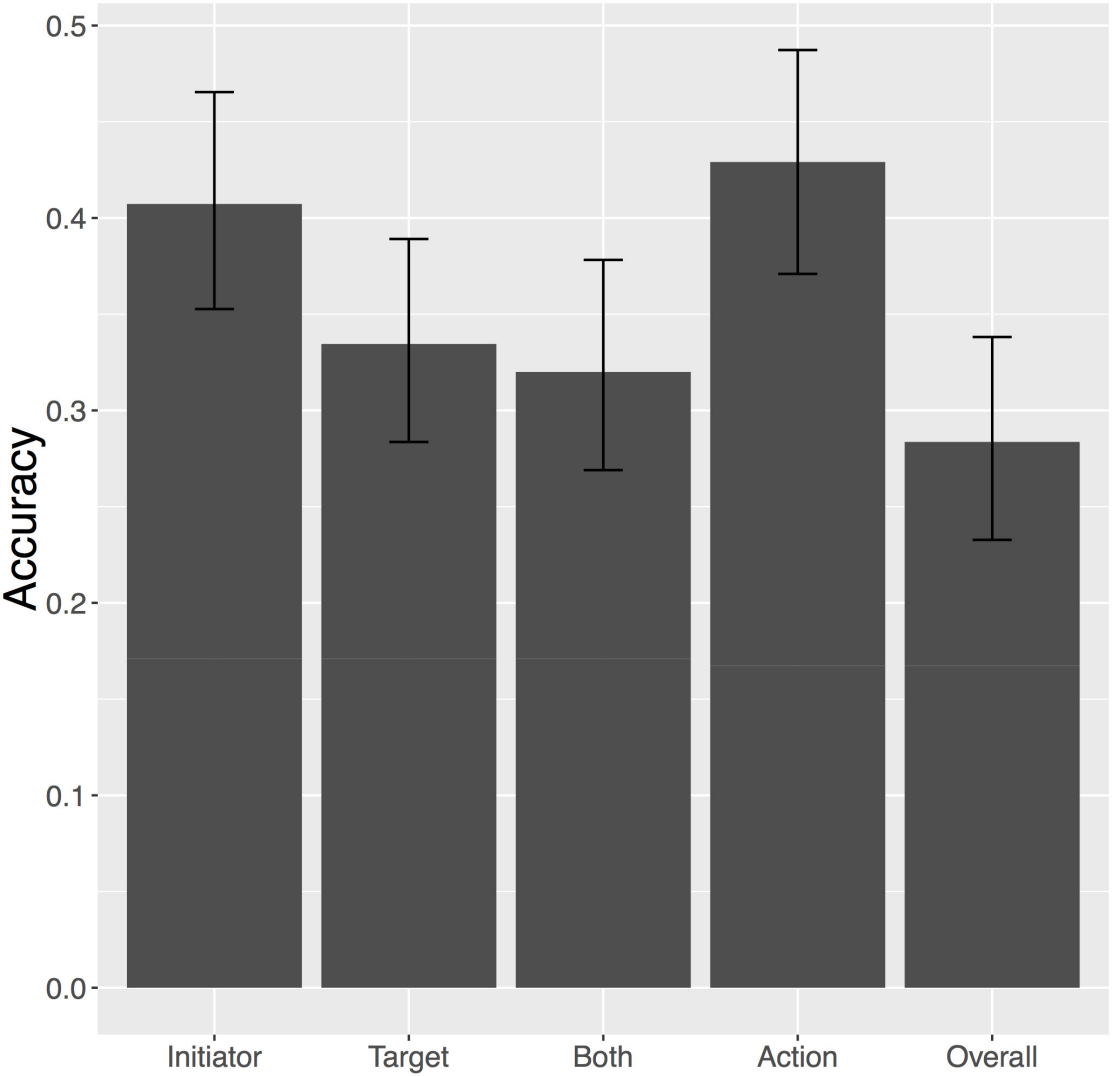
Tabari Results. Figure displays Tabari’s accuracy for the 275 documents it could code. Accuracies are reported for initiator, target, both initiator and target, action, and for the combination of initiator, target, and action. 95 percent confidence intervals are calculated from 1000 bootstrapped samples of the data.

The accuracy for the 275 news stories that Tabari could code is displayed in Fig 2. Of these 275 documents, 67 percent (183 documents) contained information about MIIs according to the gold standard. Tabari correctly identified 63 percent of those MIIs as militarized incidents. Of these 183 MIIs, Tabari correctly identified 36 percent of the initiators, 25 percent of the targets, and 39 percent of the actions. Of the 92 documents that did not contain information about MIIs, Tabari incorrectly identified 49 percent of them as containing some form of MII-related action.

We further disaggregate these results in Table 2, which provides a crosstab of the Tabari and the true (gold standard) action code for all documents. The numbers labeling each row and column in the table correspond to MII action levels. *Not MII: Domestic* indicates that Tabari coded both initiator and target as being from the same country and thus not an MII. *Not MII: International* indicates that Tabari coded an international event that is not an MII. *Null* indicates that Tabari did not produce any event data for that document.

**Table 2:** Tabari Actions.

		True Coding									
		(1)	(2)	(3)	(4)	(5)	(6)	(7)	(8)	(9)	(10)
	Null	91	1	15	3	9	4	5	20	18	9
	Not MII: Domestic (1)	28	1	5	1	1		2	4	4	10
	Not MII: International (1)	18	1	14				2	6	11	5
	Threat to use force (2)										
	Show of force (3)	1		3	1						
	Alert (4)				1						
Tabari	Mobilization (NA)				1	3				1	1
	Border fortification (5)										
	Border violation (6)										
	Occupation of territory (7)						1	3		1	
	Seizure (8)	9							5		1
	Attack (9)	28		13	2	1			1	45	10
	Clash (10)	7		1			1			5	15

**Note:** The “null” category pertains to documents where Tabari did not record any event occurring. The “domestic” category pertains to documents Tabari did not classify as taking place between two state actors. Empty cells are analogous to zeros and indicate that no documents were classified in a particular category. Column names: (1) Not MII (both Domestic and International); (2) Threat to use force; (3) Show of force; (4) Alert; (5) Border fortification; (6) Border violation; (7) Occupation of territory; (8) Seizure; (9) Attack; (10) Clash.

The bulk of correct codings came from the 137 true negatives, the 45 true *attacks*, and the 15 true *clashes*. The true negatives include the 91 *nulls* that were not MIIs, the 28 *domestic* events that were not MIIs, and the 18 *international* events that were not MIIs. The fact that most of the accuracy was due to true negatives, *attacks*, and *clashes* may be indicative of shortcomings in using Cameo to classify types of international conflict.

Tabari has greater difficulty discerning the correct actors than it does the correct action. On the full dataset, Tabari classified the initiator correctly 45 percent of the time and the target correctly 40 percent of the time. When *nulls* are dropped, the initiator accuracy is just 41 percent, while the target accuracy is 33 percent. Action accuracy, on the other hand, is 46 percent if the nulls are included, and 43 percent if nulls are removed. Finally, overall accuracy, meaning initiator, target, and action are coded correctly, is 38 percent if nulls are included, and 28 percent if they are removed. Clearly, Tabari is not effective in coding complicated social phenomena such MIIs in this manner.

Note that these findings are not incompatible with previous work that found machine coding produces data as accurate as that produced by human coders [40, 41]. In previous applications, machines and humans were compared in their ability to produce events data using the Cameo ontology. Instead, we evaluate our ability to use machine coders and the Cameo ontology to reproduce MIIs. Nevertheless, these results suggest that machine classification does not produce sufficiently accurate results to reproduce data on complex constructs such as the MII.

## Crowdsourcing

We describe a new method that supplements crowdsourcing with computational tools in an effort to obtain high accuracy *and* efficiency. The findings show that the crowdsourcing-based approach yields an actor accuracy of 76 percent, an action accuracy of 73 percent, and an overall accuracy of 68 percent. The method incurs some financial costs—about 0.62 that of experts—but is also much faster than experts and opens up useful avenues for future research. In the following section we describe the general crowdsourcing architecture used for the coding of MIIs. The IRB at Penn State approved this research. The ID is 42955.

### Crowdsourcing Architecture

Before distributing the news documents to workers, natural language processing tools were used to extract metadata from each, including the date, title, and named entities. In this case, named entities refer to relevant country names, groups, or individuals described in the document. The list of named entities are contained in Phil Schrodt’s CountryInfox.txt file at https://github.com/openeventdata/CountryInfo. These metadata were implanted into the questionnaire through an external web service with the goal of limiting user error.

Next, individuals were asked to read a news story and were asked a line of objective questions structured as a question tree. That is, their answers were used to determine which question they were asked next. The full questionnaire is available as part of the supporting materials in S1 File. The set of responses were then compiled into the crowd’s assessment of each document and were compared to the gold standard.

The questionnaire was hosted on Qualtrics, a web-based survey platform, and workers were recruited through Amazon’s Mechanical Turk (AMT), a widely used crowdsourcing platform. We utilize AMT because of its large workforce and its straightforward, flexible interface. The size of the AMT worker population and typical task length means that, even if crowdsourcing becomes a widely utilized data-collection method, a significant increase in available tasks likely would not diminish the speed or efficiency of the process. To protect the anonymity of respondents, we do not collect any demographic data, such as ethnicity or socio-economic status as part of this process.

When a worker first completed a questionnaire she was required to take a quality-control test. Such tests minimize errors, but reducing false positives usually comes at the cost of increasing false negatives and vice versa. We prioritized minimizing the occurrence of false negatives (a story about a MII classified as a non-event) because it is easier to remove false positives post hoc than it is to recover true positives that were incorrectly screened out. With this in mind, the test required the worker read and correctly classify a story about a clash between opposing military forces. Workers that failed this test were not allowed to proceed to the questionnaire. An alternative approach aimed at reducing false positives could have provided workers with a news story that did not contain information about a MII.

After passing the test, the worker was given a set of simple instructions asking her to use only the information in the story and to focus only on actions taken by countries against other countries, not non-state actors. She was then prompted with a link to a story that is hosted on an external server. For example, below is an excerpt from one of the stories:

Two Russian helicopters violated Georgian airspace in the area of the Georgian-Abkhaz border on Thursday, the Georgian Interior Ministry has reported. “The Russian MI-8 and MI-24 helicopters flew over the villages of Tanmukhuri and Khurcha, Zugdidi region, where Interior Ministry troops are stationed, at 11:00 a.m. on March 5,” says the report [42].

The worker then proceeded through the questionnaire. Upon completion, the worker was paid fifty cents plus a twenty-five cent bonus if she was determined to have answered “well.” Specifically, “well” means if the worker answers a random test question correctly, spends sufficient time on the questionnaire to convince us he or she is not randomly clicking, and writes at least three words when asked to summarize the document. Workers are made aware of this incentive structure, but not the evaluation criteria.

After reading the news story, workers were asked to extract the initiating and target actors. For example, a mockup of the questions pertaining to actors is shown below. Consistent with the example story, the named entity recognition tools identified “Russia” and “Georgia” as countries identified in the news document and provided them as options for workers to select as answers to these questions. Had other countries been referred to in the text, these too would have been offered to the workers as choices.

**What country or countries (or their military personnel) initiated the action? If civilians or non-state actors initiated an action, focus instead on the action a country took in response.**
  □ Russia
  □ Georgia
  □ Other, please enter the name of the initiating country: [__________]
**Who is the target of the action by the country/countries selected above? The target may be a country, its civilians, its territory, or a non-state actor operating within the country. Either way, please name the country that the action has been directed against.**
  □ Russia
  □ Georgia
  □ Other, please enter the name of the target country: [__________]

Workers were then asked about the events that took place. The questionnaire proceeded from questions about the most hostile event types (uses of force) to the least hostile event types (threats to use force). Upon a categorization of action type, workers were presented with the MID project’s operational definition and asked if their categorization was consistent with this definition. For example, if the worker coded the story as a show of force, as the example story should be coded, and coded the actors as Russia and Georgia, they would have been prompted with the following:

**Russia engaged in a show of force against Georgia. A show of force is defined as a public demonstration by a state of its military forces intended to intimidate another state but not involving actual combat.**
  **Examples include non-routine military maneuvers and military exercises, naval patrols immediately outside the territorial waters of another state, and the intentional violation of another state’s territorial waters or air space. Is this an accurate description of the event?**
  □ Yes
  □ No
  □ Yes, but the actors are reversed

This question is used as a final validation of the respondent’s coding and as a means of providing respondents with an opportunity to holistically review their coding of an event. If respondents answered “No” to this final validation question, they were prompted to complete the questionnaire again until they achieved a coding that they felt accurately records their conceptualization of the event. We utilize responses only where the worker answered “Yes” or “Yes, but the actors are reversed” to this question.

### Crowdsourcing Costs

In total, the questionnaire was completed 3,899 times for the sample of 446 stories. 1,644 individuals took part in this exercise, coding, on average 3.7 stories (Range: 1–55, mode: 1, i.e., 1,251 workers completed the questionnaire only once).

Of the 3,899 responses, 111 were removed because of processing issues or because respondents answered “No” to the verification question. This resulted in 46 stories having less than nine annotations (Range: 6–10, average: 8.47). These incomplete stories were excluded from the analysis to facilitate a comparison of reliability of the methods as it relates to redundancy. Some individuals completed the questionnaire for multiple stories.

The average length of time spent taking the survey was 13.37 minutes, as measured from the time a task was accepted and completed on AMT. Together, the workers completed a total of about 851 hours of work. As previously stated, each worker was paid between 50 and 75 cents depending on whether the respondent answered a bonus question correctly, provided an adequately long summary of the news document, and spent sufficient time on the survey. With this incentive structure in place, procuring 3,899 responses had the potential to cost between $1,949.50 and $2,924.25, depending on the proportion of respondents that received the 25 cent bonus. In actuality, the costs came to $2,819.5, with about 90% of workers receiving the bonus.

As expected, crowdsourced labor is considerably cheaper than that obtained from highly trained experts. While individuals recruited online worked for about 3.31 dollars an hour, the expert coders were paid $15 per hour, representing relatively typical wages for trained research assistants. Moreover, the expert coders classified news documents at a similar rate to the crowd, indicating that the former are not necessarily more economical in their use of time. Of course, experts are expected to produce more accurate classifications than the average crowd worker, and fewer coders are required to classify each news document as a result. Even so, employing three expert coders to classify each of the 446 documents required roughly 300 hours of work, costing about $4,500, considerably more than that spent on the crowdsourced labor.

Although widely used, ethical and legal questions about the wages workers earn and the relationship between the requester and the worker remain [43]. While these questions are beyond the scope of this article, our estimated hourly wage of $3.31 (including the bonus) is above the mean of $1.25 reported in [44], and it is above the mean of $2.30 for US workers reported in [45]. Furthermore, since the hourly wage is calculated using the time from start to finish, our true hourly wage is likely higher. For example, if a worker begins, pauses, and continues at a later time, the hourly wage will be deflated. In any case, the marketplace for crowdworkers helps to ensure fair market wages, as it displays average time to complete a task, and a lower bound of compensation to be expected.

More than the hourly or aggregate economic costs, the greatest advantage of crowdsourced data collection is the relative gains in terms of the time required for data collection. While the crowd completed a total 851 hours of work, the time between the initial posting of this job and its completion was roughly five days. We posted job requests in intervals during this time in order to monitor crowd responses. Had we posted the full set of job requests at once, this process would have been completed considerably faster. By contrast, it took three expert coders nearly five weeks to complete the same task, working an average of 20 hours per week. Thus, the core advantage of crowdsourcing in this application is not necessarily monetary gains, but time saved till task completion. Even if online workers were paid at the same rate as expert coders, the former would still be capable of producing quality data far more quickly than the latter due to the highly parallelized nature of crowdsourced methods. Furthermore, crowdsourcing is scalable; as the task becomes larger, one may simply recruit more workers to participate. In short, as has been consistently found in other applications, crowdsourcing yields significant gains in both the costs and time required to complete tasks typically left to expert coders.

### Crowdsourcing Accuracy

To improve accuracy and leverage the size of the crowd in data collection, the same story was analyzed by multiple workers. Like any crowdsourcing approach, this poses a problem of aggregation: how can the output from several workers be combined most effectively? A simple technique is to code the MII attributes based on a plurality vote, which we termed *naive voting*. Although the naive voting aggregation is effective, it is also limited in that it aggregates individual MII characteristics in isolation, rather than utilizing the full spectrum of information about the MII provided by respondents. For example, when identifying the initiator state through naive voting, only classifications of the initiator state were used, ignoring other information provided by the respondent that may aid in correctly identifying the initiator.

Following [9, 46] and others, we utilized a more advanced aggregation technique to improve upon the naive voting approach. Specifically, we used Bayesian network models to leverage the full range of information provided by respondents about the MII [47]. We termed this *error corrected voting*. The analysis was conducted using the Waikato Environment for Knowledge Analysis (WEKA 3.7.10). We used the Bayesian network because it outperformed other general machine learning algorithms [48], including support vector machines and random forests, as well as more specialized crowdsourcing aggregation algorithms such as ZenCrowd [49]. In the error corrected voting algorithm, each MII attribute was *predicted* using a Bayesian network model. The predictors were the individual worker’s responses to each question. The model’s predictions for each MII attribute were then aggregated using the same voting procedure as with the naive voting method.

#### An Application of Bayesian Networks

A Bayesian network is a machine learning classifier based on a probabilistic model that represents probabilistic variables in a directed acyclic graph. In this way, it leverages dependencies between variables to uncover meaningful relationships. Here, each node in the network represents a question asked to the workers, and each connection between nodes represents a probabilistic dependence between those questions (see [48] for technical details). As such, stronger connections correspond to stronger or more clearly identifiable relationships. Relative to other commonly used classifiers in machine learning, this method of aggregation quick to construct, makes minimal structural assumptions about the nature of relationships in the data, and is generally robust to minor variations in the data [50, 51]. This approach is also built entirely from data provided by questionnaire responses (*training data*), with no outside influence or information, making the approach very flexible to changes in variables of interest or questionnaire design.

“Error correction” refers to the notion that the Bayesian network model utilizes the worker’s responses to construct a network that describes dependencies among the questions. This network can then generate accurate predictions pertaining to the initiators, targets, and actions within each story. This is done by first constructing an initial hypothesis about the network of dependencies in the data and then “correcting” this network by examining a set of alternative hypotheses or dependencies about the network structure. The dependencies between variables uncovered through this process are then used to generate weighted decisions pertaining to each variable of interest. For example, a decision rule might be a probabilistic variant of “IF Action Type == Show of Force AND Initiator State == North Korea THEN Target State = South Korea.” This hypothetical rule might be a function of the fact that an overwhelming proportion of North Korean shows of force are directed at South Korea, and so the target state in such scenarios is largely assured.

An additional strength of this approach in this application is that it can utilize information from auxiliary and redundant questions asked to each respondent, which increases the breadth of information available for network construction. The auxiliary questions asked about important, but peripheral characteristics of the MII, such as the presence of non-state actors in the story or fatalities documented in the story. The redundant questions were more general, asking simply for broad classifications of the news event, such as whether the action in the story was cooperative/conflictual in nature and whether the action was material or verbal in nature. Using these additional questions allows the potential to increase accuracy by increasing the scope of information exploited to generate predictions.

Note that the model, like any machine learning classifier, does not only identify relationships in the worker responses to the questions. It also allows for error-correction, as it identifies answers that typically co-occur. For example, it may learn which countries share borders, and which countries have been involved in conflicts. This way, it improves overall performance by acquiring geopolitical knowledge.

Like any statistical model, it is necessary to ensure that the Bayesian network model is not overfit to idiosyncrasies within the training data. To ameliorate this risk, we automatically trained many versions of the model, each from a random subset of the data and examined for accuracy using the data that were not used for training (such *cross-validation* is a standard approach in machine learning applications). This helps ensure that the reported accuracy is robust and generalizable to future data, rather than developing decision rules based only on select cases. Note that application centers on international conflict processes over a relatively short period of time (2007–2010). Because of this, a single training sample is drawn from the population of news stories.

However, it is a limiting property of the machine learning approach that it learns and exploits knowledge of geopolitical relations. This will naturally change over time. Thus, a classifier should not be trained on data from one time period, and then applied to data from a very different one. Instead, training on annotated samples from the target time period should address such concerns. Additionally, inclusion of further, multi-modal features that, for example, describe geographical proximity between actors, may help the model generalize better.

#### Actors

**Fig 3:**
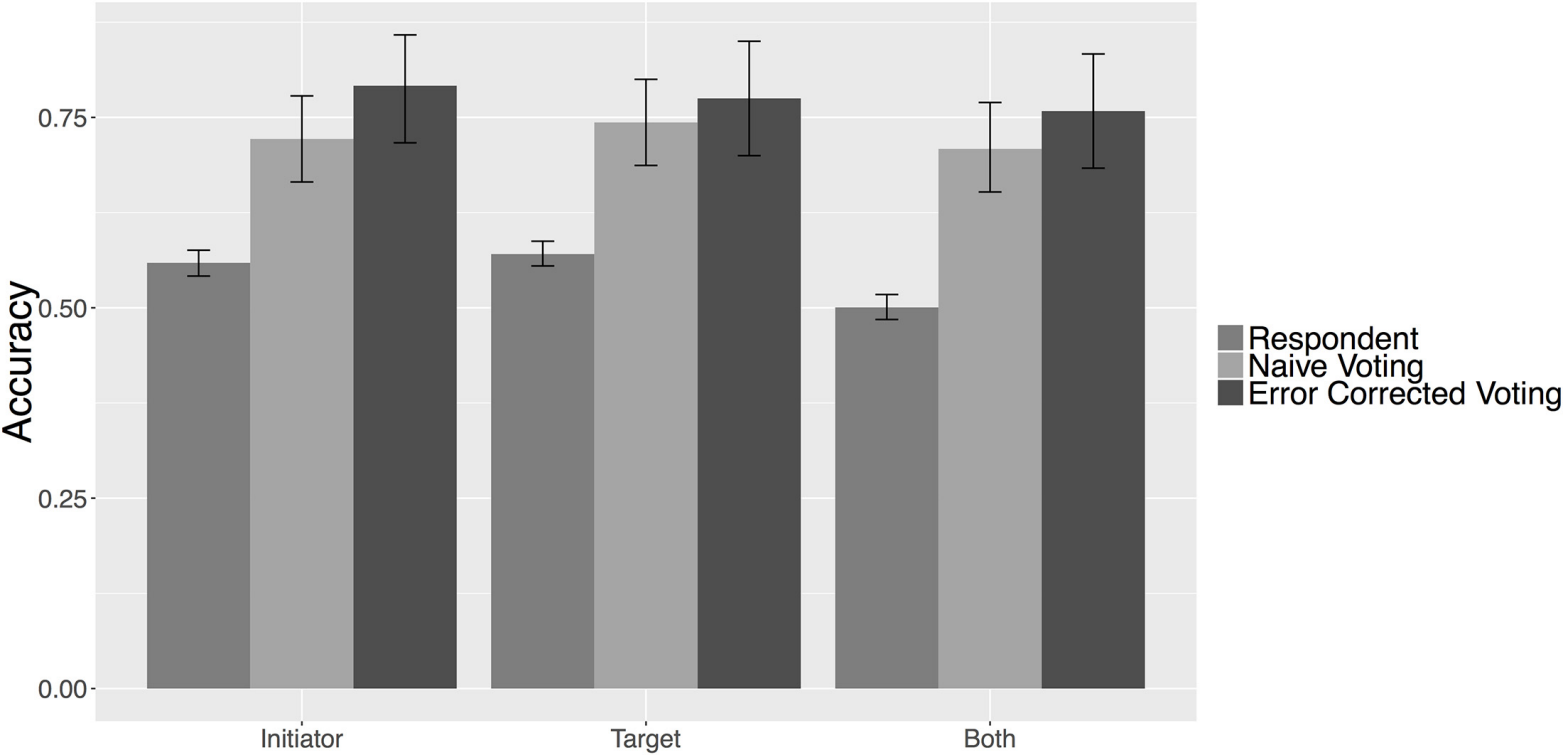
Crowdsourcing Actor Accuracy. Figure displays crowdsourcing accuracy in identifying the state actors described across the sample of news documents. 95 percent confidence intervals are calculated from 1000 bootstrapped samples of the data.

Fig 3 reports the crowdsourcing accuracy in classifying the actors in each news story. As was the case when computing accuracy using machine coding, we consider the classification of actors to be correct for all cases that respondents *correctly* classify news documents as non-MIIs, since there are no initiators or targets in these cases. These results are reported at both the respondent and group level, with the latter calculated using both naive and error corrected voting. We find that, while individual respondents may preform relatively poorly, accuracy is improved after aggregating these responses. We also find that *error corrected* voting modestly improves overall accuracy when compared to the more rudimentary *naive voting* form of aggregation. These results are important and underscore two fundamental strengths of crowdsourced data collection. First, while individual classifications of the data may be inaccurate, the size of the crowd can be leveraged to dramatically improve the quality of the data and, as Fig 3 illustrates, even straightforward aggregation techniques offer substantial improvements in accuracy. Second, crowdsourcing allows researchers flexibility in choosing aggregation methods that may be used to further improve the quality of results.

The crowdsourcing method identified actors with relatively high accuracy. Specifically, using the error corrected voting method, we correctly identified the initiator 79 percent of the time, the target 78 percent of the time, and both initiator and target 76 percent of the time. These results are a substantial improvement over the accuracy reported for the machine coding approach, which only ranges from 38 to 45 percent in actor categories.

We also examined whether there are any systematic causes of error among respondents. We found that a substantial number of responses with incorrect actor codings were from news documents about non-state actors such as rebel groups and terrorist organizations. Respondents often incorrectly identified these instances as MIIs, even though they do not take place between two state actors, as required by the coding rules. Respondents tended to misclassify the relevant actors for these news documents as a result. This bias is discussed in more detail below.

Another, less systematic source of error occurred when respondents provided a free-text entry too specific for the coding classification, referencing specific individuals or villages instead of countries. In a real-world application, such errors might be handled by post-processing responses to map the free-text entry to the corresponding state.

#### Actions

In this section we report accuracies for incident occurrence, hostility level, action type, and the overall accuracy. Incident occurrence is a dichotomous measure of whether the news story presents some type of MII. Hostility levels refer to the broader categories or groupings of militarized actions laid out by the MII coding rules. The five hostility levels are *No Militarized Action*, *Threat to Use Force*, *Display of Force*, *Use of Force*, and *War*. Overall accuracy refers to the correct coding of both actors and action type.

**Fig 4:**
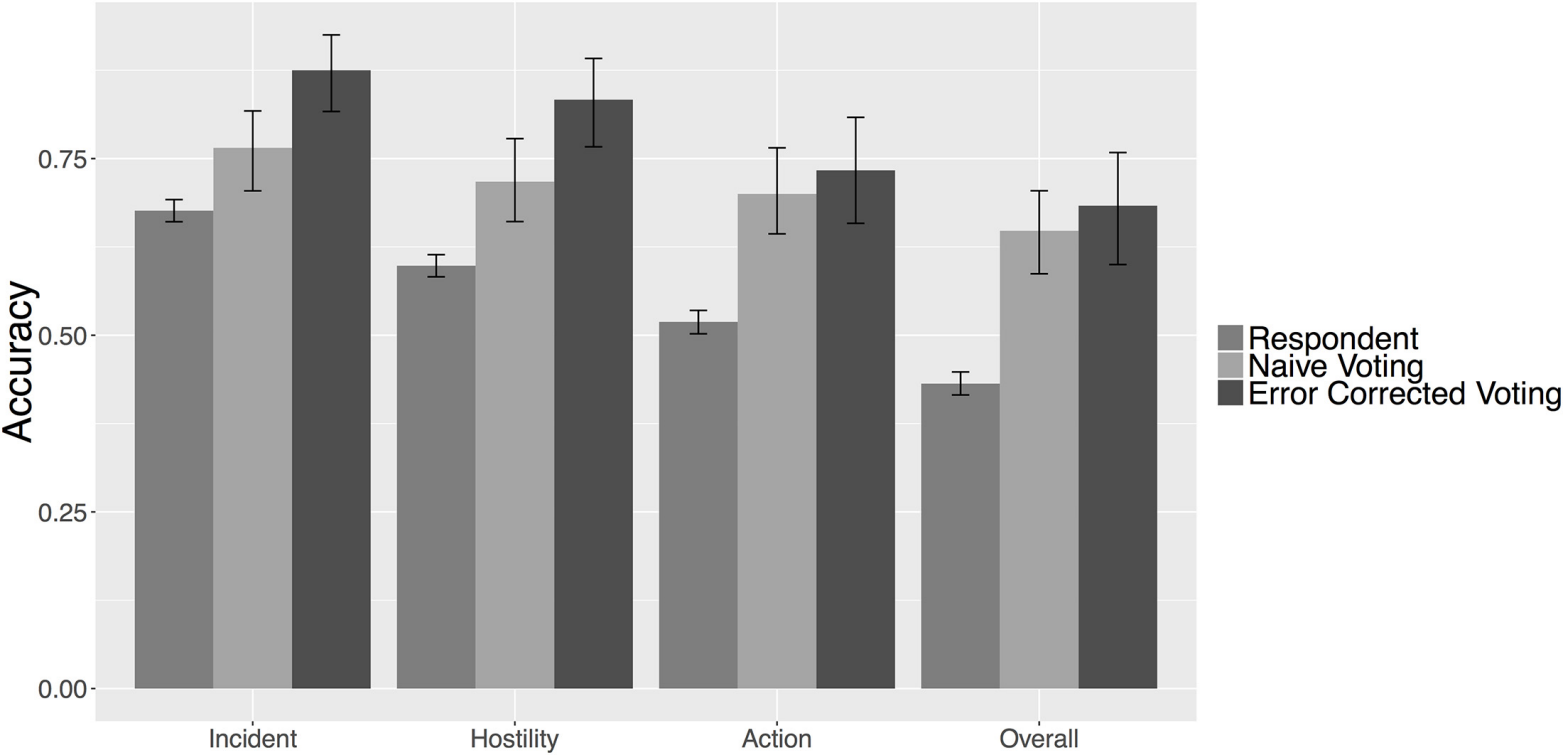
Action Accuracy. Figure displays crowdsourcing accuracy in identifying the militarized actions described across the sample of news documents. 95 percent confidence intervals are calculated from 1000 bootstrapped samples of the data.

Exploring Fig 4, the incident accuracy measures how well the crowd is able to distinguish between MIIs and non-MIIs. For voting methods, where there is a tie one answer is chosen at random. At the respondent level, the incident accuracy is just 68 percent, but it increases to 77 percent using *naive voting* and 88 percent using *error corrected voting*. The crowd performs well when distinguishing hostility levels, and these results, too, see significant improvements using advanced techniques. The hostility accuracy is 60 percent at the respondent level, 72 percent using *naive voting*, and 83 percent using *error corrected voting*. The action accuracy measures the ability of the crowd to correctly identify the correct MII action type. At the respondent level, this is just 52 percent, but the accuracy increases to 70 percent when using *naive voting* and 73 percent using *error corrected voting*.

Table 3 reports the cross tabulation of the crowd’s responses (using *naive voting*) against the expert coding with respect to each specific action type. Note that while Table 3 reports *Tie* as a separate category, the following results are reported when one of the tied categories are chosen at random. In total, expert coders classified 235 news stories as containing information about MIIs and 165 news stories as non-incidents. At the incident level, of the 235 MII stories, the crowd classified 211 correctly and 24 incorrectly. When these false negatives occur, the crowd often mistakes shows of force, seizures, and attacks for non-incidents. Of the stories containing no information about MIIs, the crowd classified 104 correctly and 61 incorrectly. Among these false positives, the crowd is most likely to classify non-events as attacks. At a rate of about five to two, the crowd clearly produces more false positives than false negatives. This is consistent with our previously-stated intent to structure a survey that minimizes the misclassification of stories about MIIs.

**Table 3:** Crowdsourcing MII Actions: Naive Voting.

		True Coding									
		(1)	(2)	(3)	(4)	(5)	(6)	(7)	(8)	(9)	(10)
	Not MII (1)	101		5	1	2			7	5	1
	Threat to use force (2)	3	3								
	Show of force (3)	4		26		1					
	Alert (4)										
	Border fortification (5)	1		3	1	6					
Crowd	Border violation (6)	1			1	1	1				
	Occupation of territory (7)	1		1		1	2	2		1	
	Seizure (8)	2						3	22		
	Attack (9)	30		3			1	1		65	4
	Clash (10)	7		2	3					3	33
	Tie	15		6	2	2	1	4	3	4	2

**Note:** The “tie” category pertains to documents for which respondents were equally divided in classifying a document among two or more action types. Empty cells are analogous to zeros and indicate that no documents were classified in a particular category. Column names: (1) Not MII; (2) Threat to use force; (3) Show of force; (4) Alert; (5) Border fortification; (6) Border violation; (7) Occupation of territory; (8) Seizure; (9) Attack; (10) Clash.

Moving beyond these aggregate classifications, Table 3 also shows that non-experts struggle when identifying the third hostility level, *Display of Force*. With respect to *Displays of Force*, many *shows of force* are miscoded, and the distribution of these false codings are dispersed across five different action types. This suggests that non-experts do not understand what constitutes a *show of force*, which may reflect ambiguities in the coding rules or perhaps loose wording in the questionnaire. For example, a *show of force* is “a public demonstration by a state of its military forces intended to intimidate another state but not involving actual combat operations” [52, 5]. Contrast this with an *attack*, which is “the use of regular armed forces of a state to fire upon the armed forces, population, or territory of another state” [52, 6]. Conceptually, it is more difficult to observe the intent to intimidate than it is to observe a state military firing upon another state. This indicates that the accuracy attained when using non-expert data collection is partially dependent on the ease of identifying distinctions in the concept of interest. Thus, some concepts will lend themselves to this method more easily than others.

The overall accuracy, which measures both actions and actors, represents the crowd’s ability to construct an MII. To do this, respondents must correctly identify the *Initiator State*, the *Target State*, and the *Militarized Action*. At the respondent level, the overall accuracy is just 43 percent. However, using *naive voting* this accuracy increases to 65 percent, and increases to 68 percent when using the *error corrected voting* model.

Table 4 shows the cross tabulation of the crowd’s responses against expert coding when the error corrected voting method has been applied. While the accuracy is slightly improved using the error corrected method, as shown in Fig 4, there are meaningful differences in their classifications that should be explored

**Table 4:** Crowdsourcing MII Actions: Error Corrected Voting.

		True Coding									
		(1)	(2)	(3)	(4)	(5)	(6)	(7)	(8)	(9)	(10)
	Not MII (1)	104		1	1			1		3	1
	Threat to use force (2)	2	3								
	Show of force (3)	19		35	3				1	2	
	Alert (4)	2		1							1
	Border fortification (5)	3				10		1			
Crowd	Border violation (6)							1			
	Occupation of territory (7)	2				2	1	6	2		
	Seizure (8)	8		2					29	1	
	Attack (9)	16				1	3	1		55	2
	Clash (10)	6		6	4		1			17	34
	Tie	3		1							2

**Note:** The “tie” category pertains to documents for which the Bayesian network predictions were equally divided in classifying a document among two or more action types. Empty cells are analogous to zeros and indicate that no documents were classified in a particular category. Column names: (1) Not MII; (2) Threat to use force; (3) Show of force; (4) Alert; (5) Border fortification; (6) Border violation; (7) Occupation of territory; (8) Seizure; (9) Attack; (10) Clash.

Using error corrected voting, there are only 6 documents classified as a *Tie*, while with naive voting there are 39. Therefore, on the whole, categories other than *Tie* and more likely to be predicted. However, not all categories have increased, and of those that have, not all have increased proportionately. Predictions of *shows of force* almost doubled, from 31 with naive voting to 60 with error corrected voting. Accuracy for this category remains a problem; with naive voting 20 true *shows of force* were classified as something else, and with error corrected voting 25 predicted *shows of force* were incorrect. This action type remains difficult to classify.

Another interesting difference is in the *not MII* category. Using naive voting, 122 documents were predicted as *not MII*, of which 101 were correct. With error corrected voting, only 111 were predicted as *not MII*, of which 104 were correct. Considering our desire to limit false negatives (it is less harmful to incorrectly classify a document as an MII than to incorrectly classify a document as not an MII), this is an improvement over the naive method.

The *attack* and *clash* categories present another meaningful difference in the two aggregation procedures. Using naive voting, the *attack* category was overpredicted (78 true, 104 predicted), as was the emphclash category (40 true, 48 predicted). However, with error corrected voting the *clash* category is heavily overpredicted (40 true, 68 predicted) and the *attack* category is predicted at a proportional rate (78 true, 78 predicted). In other words, using error corrected voting a document is less likely to be predicted as an *attack*, and more likely to be predicted as a *clash*. However, the proportional rate does not mean a higher accuracy–only 55 of the predicted *attacks* are correct, while for naive voting 65 are correct. This loss in accuracy is not compensated in the *clash* category, where 34 predicted *clashes* were correct, while for naive voting 33 were correct.

Overall, the results of our crowdsourcing method reveal (and confirm) several interesting insights. First, as is typically the case in crowdsourcing applications, we find that while individual workers produce noisey or innacurate data pertaining to MIIs, the wisdom of the crowd can be leveraged to produce accurate data in the aggregate. The results of the naive voting aggregation show that, while more sophisticated aggregation methods may perform better, even simple aggregation techniques provide substantial increases in the accuracy of collected data. Furthermore, different aggregation methods will produce different types of error. For our purposes, the gains from the Bayesian network are large for *Incident* prediction, which is complementary to our desire to limit false negatives. Nevertheless, this is simply one means of aggregating responses. Practitioners would be well suited to exploring alternative or supplementary strategies of aggregation to determine which is best suited toward a particular line of research [53]. Thus, we see future research incorporating machine learning algorithms as beneficial for this project. Finally, as shown in the dispersion of *show of force* judgments, crowdsourcing appears to lend itself well to some concepts and not as well to others. This finding holds for both the naive and error corrected voting methods. For nuanced categories such as *show of force*, more input from the crowd and more informative questions are likely necessary to improve accuracy levels. Alternatively, an approach might be taken where crowdsourced output is used to identify subsets of stories that would benefit most from expert evaluation.

## Conclusion

Incumbent methods of data collection often force researchers into a dilemma in which they must choose between cost and accuracy. While utilizing small groups of expert coders provides accurate data, the process is slow and expensive. Conversely, automated machine coding methods are cheap and fast, but consistently produce noisy data. With respect to our application to conflict data, the results presented here show that a third approach, one that merges crowdsourcing and automated techniques, marginalizes these drawbacks while retaining select benefits from each.

Our comparison of crowdsourced data collection methods to machine coding suggests that the former is better able to reproduce expert classifications according to the MII coding rules. Focusing on three important and basic characteristics of the MIIs, non-expert workers identified and classified the actors and militarized actions in a series of news documents at between 68 and 76 percent accuracy. Machine coding, on the other hand, produced accuracy measures that range from 30 to 45 percent. Of course, this is but one application comparing the relative advantages of crowdsourcing and machine coding.

The fact that crowdsourcing performed so well in this application is particularly significant given that the MII is a subjective construct based on several layers of coding rules. While MIIs are complex constructs, the development of clear and straightforward questionnaires allow non-expert crowds to recreate such data at relatively high accuracy. These results robustly support the notion that, in the aggregate, non-experts can largely supplant experts for simple data collection, even for concept-driven data structures.

Our analyses also demonstrate novel ways in which crowdsourcing can be interlaced with other methodological advancements. Pre-processing news documents to extract named state entities allowed us to provide workers with valid options when identifying actors in associated documents, which prevented incorrect or incoherent results. Equally important, we find that Bayesian modeling techniques can be used to more accurately aggregate responses obtained from multiple respondents.

Crowdsourcing also greatly reduced the time and financial costs and generated data much faster than data collected by small numbers of expert coders. The gains in time are particularly consequential, as reliance on human labor represents a primary bottleneck in many data collection processes (e.g., [5]).

This project provides a baseline analysis of the utility of crowdsourcing for collecting conflict data and serves as a springboard for further exploration and improvements in this burgeoning method of data collection. The results we uncover point toward three viable areas of research in the study of conflict data collection. First, while we find that crowdsourcing outperforms purely automated methods in classifying MIIs, it is not yet known whether this result extends to measurement tasks relating to civil conflict, mass atrocities or similar concepts. Second, future work is needed to more fully explore viable means by which machine coding and computational methods can be synthesized with crowdsourcing techniques when classifying political events. Third, though Bayesian methods appear to improve accuracy in aggregating crowdsourced responses, this is but one of many possible aggregation strategies. Complex dependencies often exist between political actors and events, and the nature of these dependencies are prone to change over time. Future work might explore how adaptive or dynamic modeling techniques can be fruitfully applied when aggregating human-coded classifications of such events. Probing these issues further will be critical in developing a stronger understanding of measurement strategies available to political science researchers.

## Supporting Information

S1 File Full appendix. This pdf file contains all supporting information, including tables, figures, the crowdsourcing questionnaire, and additional details.
(PDF)
