# Supplementary material for: Crowdsourcing the Measurement of Interstate Conflict
Source: PLoS One. 2016 Jun 16;11(6):e0156527. doi: 10.1371/journal.pone.0156527 (PMC4911154; doi:10.1371/journal.pone.0156527)
Supplement: S1 File — This pdf file contains all supporting information, including tables, figures, the crowdsourcing questionnaire, and additional details. (PDF) [file pone.0156527.s001.pdf]

Crowdsourcing the Measurement of Interstate Conflict: Supporting Information

Vito D'Orazio[1,*,◕], Michael Kenwick[2,◕], Matthew Lane[2,◕], Glenn Palmer[2,◕], David Reitter[3,◕],

1 School of Economic, Political, and Policy Sciences, University of Texas at Dallas, Richardson, TX, USA
2 Department of Political Science, Pennsylvania State University, University Park, PA, USA
3 College of Information Sciences and Technology, Pennsylvania State University, University Park, PA, USA

◕These authors contributed equally to this work.
*** dorazio@utdallas.edu**

Contents

1 Analysis of Automated Machine Classification 2

2 Crowdsourcing 4
2.1 Crowdsourcing Architecture 4

3 Additional Crowdsourcing Analyses 7
3.1 Coding with Undergraduates 7
3.2 Geolocation of Respondents 8
3.3 Crowdsourcing and Resource Allocation 10

4 Crowdsourcing Questionnaire 12

1 Analysis of Automated Machine Classification

To machine code the documents, we used Tabari [1] and the Conflict and Mediation Event Observations (Cameo) ontology [2]. We used Tabari version `TABARI.0.8.4b2` and Cameo verbs version `CAMEO.091003.master.verbs`. The actors files used are: `nouns\_adj\_null.110124.txt`, `Phoenix.Countries.140130.actors.txt`, `Phoenix.Internatnl.140130.actors.txt`, and `Phoenix.MNSA.140131.actors.txt`. The agent file is `Phoenix.140127.agents.txt`, and the options file is the default `pipeline.options.txt`. All software has been retrieved from `https://github.com/openeventdata`.

Tabari produced a database of event data that detailed which actor initiated an action, which actor was the target of this action, the CAMEO action type, and the date on which the event occurred. To discern the codeable MII attributes from this dataset, each CAMEO action type was mapped onto an MII code using a deterministic set of rules. These rules pertain to both the actors and the actions.

With respect to the actors, MIIs occur, by definition, between two or more countries. Therefore, when a Tabari coded event contained actors from the same country, we considered this to be a *domestic* event and therefore *not an MII*. Otherwise, the event was *international* and thus *potentially an MII*.

For all potential MIIs, the Tabari coded action was mapped to an MII code, as in Table A. For example, if an action was coded by Tabari as *use unconventional violence*, it was mapped to an MII *attack*. The actions that did not have an MII counterpart, such as *meeting*, were coded as *not an MII*. For each story, if more than one event was coded by Tabari, the highest level MII action was used. If Tabari coded two *attacks* in the same document but with inverted actors, we considered this to be a *clash*. For example, a document that is coded twice as "Fight with small arms and light weapons," once PAK $\rightarrow$ IND, and once IND $\rightarrow$ PAK, is a *clash*.

Of the 450 documents, 175 were *null*, which indicated that Tabari did not code any event data for the document. A *null* can be produced if no CAMEO event took place within a particular news document, if the document lacked the specific verbiage required for Tabari to map a story to a particular action type, or simply as the result of technical issues such as a failure to correctly parse complex sentence structures. For

Table A in S1 File: Mapping Action Types from CAMEO to MII

CAMEO Action Type	CAMEO Code	MII Action Type	MII Code
Threaten with military force	138	Threat to use force	1
Threaten blockade	1381	Threat to blockade	2
Threaten occupation	1382	Threat to occupy territory	3
Threaten unconventional violence	1383	Threat to use force	1
Threaten conventional attack	1384	Threat to use force	1
Threaten attack with WMD	1385	Threat to use CBR weapons	5
Exhibit force posture	15	Show of force	7
Demonstrate military or police power	150	Show of force	7
Increase military alert status	152	Alert	8
Mobilize or increase police power	153	Show of force	7
Mobilize or increase armed forces	154	Mobilization	10
Seize or damage property	171	Seizure	15
Confiscate property	1711	Seizure	15
Destroy property	1712	Attack	16
Arrest, detain, or charge with legal action	173	Seizure	15
Use tactics of violent repression	175	Attack	16
Assault	18	Attack	16
Use unconventional violence	180	Attack	16
Abduct, hijack, or take hostage	181	Seizure	15
Physically assault	182	Attack	16
Sexually assault	1821	Attack	16
Torture	1822	Attack	16
Kill by physical assault	1823	Attack	16
Carry out location bombing	1834	Attack	16
Assassinate	186	Attack	16
Use conventional military force	190	Attack	16
Impose blockade, restrict movement	191	Blockade	13
Occupy territory	192	Occupation of territory	14
Fight with small arms and light weapons	193	Attack	16
Fight with artillery and tanks	194	Attack	16
Employ aerial weapons	195	Attack	16
Employ precision-guided aerial munitions	1951	Attack	16
Employ remotely piloted aerial munitions	1952	Attack	16
Violate ceasefire	196	Attack	16
Use Unconventional Mass Violence	20	Attack	16
Use unconventional mass violence	200	Attack	16
Engage in mass killings	202	Attack	16
Engage in ethnic cleansing	203	Attack	16
Use weapons of mass destruction	204	Use of CBR weapons	19
Use CBR weapons	2041	Use of CBR weapons	19
Detonate nuclear weapons	2042	Use of CBR weapons	19

the full set of news documents, *nulls* were coded as *not an MII*. To allow for the likely case that technological improvements decrease the number of *nulls*, the results are reported for both the full document set and the subset of documents for which Tabari produced event data. When included, nulls constitute non-MIIs under the assumption that CAMEO may have correctly identified a story as containing no information about militarized events.

Coding with Petrarch

The automated analysis of this document set was also completed using Petrarch, Tabari's successor program that is still under development. We used Petrarch version 0.1a0, available at `https://github.com/openeventdata/petrarch`.

Petrarch extracted 139 events from this set while Tabari found 473. The

accuracy of TABARI was substantially better across each category. Given that PETRARCH has been released as an alpha version, and systematic comparisons between it and TABARI have not yet been conducted, we deem the TABARI results more appropriate for our analyses.

2 Crowdsourcing

Our method combines crowdsourcing and computational tools in an effort to obtain high accuracy *and* efficiency. In the following section we describe the general crowdsourcing architecture used for the coding of MIIs.

2.1 Crowdsourcing Architecture

Individuals were asked to read a news story and to answer simple, objective questions about it. The full questionnaire is available at the end of this document. Prior to initiating the questionnaire, natural language processing tools were used to extract metadata from each document, including the date, title, and named entities. In this case named entities refer to relevant state names or individuals representing the state. The list of named entities are contained in Phil Schrodt's `CountryInfo.txt` file, found at `https://github.com/openeventdata/CountryInfo`. These metadata were piped into the questionnaire through an external web service, providing the workers computationally generated information about the document to help guide their decisions.

The questionnaire was hosted on Qualtrics, a web-based survey platform, and workers were recruited through Amazon's Mechanical Turk (AMT), a widely used crowdsourcing platform. Amazon Mechanical Turk hosts many thousands of workers. We utilized this system because of its straightforward and flexible interface. Additionally, the size of the AMT worker population and typical task length means that, even if crowdsourcing becomes a widely utilized data-collection method, a significant increase in available tasks likely would not diminish the speed or efficiency of the process. To protect the anonymity of respondents, we did not collect any demographic data, such as ethnicity, geographic location, or socio-economic status. While it is perhaps conceivable that variation in such demographic markers leads to variation in

the quality of data produced through crowdsourcing, such propositions are outside the scope of this project and, as such, we leave the testing of such propositions to future research. In a following section of this appendix, however, we provide some initial analyses of whether the geo-location of respondents significantly influences the quality of the data produced by the crowd.

When a worker completed the questionnaire for the first time, she was required to take a quality-control test, essentially a shortened and easier version of the actual survey. Provided the worker passed the test, she was given a set of simple instructions asking her to use only the information in the story and to focus only on actions taken by countries against other countries, not non-state actors. She was then prompted with a link to a story that was hosted on an external server. The worker then proceeded through the questionnaire until completion, when she was given a unique code to submit to AMT for payment. Upon completion, the worker was paid fifty cents plus a twenty-five cent bonus if she was determined to have "answered well." That is, if the worker answers a random test question correctly, spends sufficient time on the questionnaire to convince us he or she is not randomly clicking, and writes at least three words when asked to summarize the document. Workers are made aware of this incentive structure prior to completing the questionnaire.

> Two Russian helicopters violated Georgian airspace in the area of the Georgian-Abkhaz border on Thursday, the Georgian Interior Ministry has reported. "The Russian MI-8 and MI-24 helicopters flew over the villages of Tanmukhuri and Khurcha, Zugdidi region, where Interior Ministry troops are stationed, at 11:00 a.m. on March 5," says the report [3].

An example story is shown above. After reading the news story, workers were asked to extract the initiating and targeted actors. For example, a mockup of the questions pertaining to actors is shown below. Consistent with the example story, the named entity recognition tools identified "Russia" and "Georgia" as countries identified in the news document and provided them as options for workers to select as answers to these questions. Had other countries been referred to in the text, these too would have been offered to the workers as choices.

> **What country or countries (or their military personnel) initiated the action? If civilians or non-state actors initiated an action, focus instead on the action a country took in response.**
>
> ☐ Russia
>
> ☐ Georgia
>
> ☐ Other, please enter the name of the initiating country: [__________]
>
> **Who is the target of the action by the country/countries selected above? The target may be a country, its civilians, its territory, or a non-state actor operating within the country. Either way, please name the country that the action has been directed against.**
>
> ☐ Russia
>
> ☐ Georgia
>
> ☐ Other, please enter the name of the target country: [__________]

Following the actor questions, workers were asked a series of questions about the events that took place between these actors. The questionnaire proceeded from questions about the most hostile event types (uses of force) to the least hostile event types (threats to use force). Upon a categorization of action type, workers were presented with the MID project's operational definition and asked if their categorization was consistent with this definition. For example, if the worker coded the story as a show of force, as the example story should be coded, and coded the actors as Russia and Georgia, they would have been prompted with the following:

> **Russia engaged in a show of force against Georgia. A show of force is defined as a public demonstration by a state of its military forces intended to intimidate another state but not involving actual combat.**
>
> **Examples include non-routine military maneuvers and military exercises, naval patrols immediately outside the territorial waters of another state, and then intentional violation of another state's territorial waters or air space. Is this an accurate description of the event?**
>
> ☐ Yes
>
> ☐ No
>
> ☐ Yes, but the actors are reversed

This question is used as a final validation of the mapping between the respondent's concept of an event and the official MID concept coding and as a means of providing respondents with an opportunity to holistically review their coding of an event. If respondents answer "No" to this final validation question, they are prompted to complete the questionnaire again until they achieve a coding that they feel accurately records their conceptualization of the event. We utilized responses only where the worker answered "Yes" or "Yes, but the actors are reversed" to this question.

3 Additional Crowdsourcing Analyses

3.1 Coding with Undergraduates

Recruiting workers on AMT is not without cost. To explore one alternative source of labor, a sample of undergraduate students were recruited to classify news stories using the same questionnaire and survey architecture developed for the workers recruited through AMT. These students were drawn from courses in international relations at two major U.S. universities. Unlike the AMT workers, these students were familiar with the concept of international conflict and were incentivized by the potential to receive extra credit in the course. Because the pool of undergraduate participants was small relative to AMT respondents, we used a random sample of 100 news stories drawn from the document set of 450. In total, 146 undergraduate students took part in the activity, providing 428 responses.

Table B in S1 File: Comparing Respondent-Level Accuracy

	Undergraduate	AMT
Initiator	61.21	58.30
Target	57.01	57.48
Dyad	63.08	63.54
Both	56.31	52.46
Action	54.44	49.21
Overall	47.43	42.76
n	428	3,782

All values are percent correct.

The respondent level accuracy of the undergraduate annotations are reported in Table B. Note that these results are reported when actors are coded as *none* when a worker codes an event as a *non-MII*. As a result, they differ slightly from those reported in the main results. In general, the accuracy of undergraduate respondents is comparable to that of workers recruited through AMT. Undergraduates are, however, more accurate when classifying the militarized actions taking place in each news document, and this difference is statistically significant. Tests of statistical significance are conducted using Welch's t-test with the null hypothesis being rejected when the p-value is less than 0.05. These results indicate that alternative, and viable, sources of labor do exist and may be leveraged to further improve accuracy when additional

workers cannot be recruited through monetary incentivization.

3.2 Geolocation of Respondents

In this section we examine the geographic dispersion of the workers employed in our study. As stated in the main document, we refrain from collecting any demographic or socioeconomic information that could be used to identify respondents. We do, however, geolocate respondents in an attempt to gain an understanding of whether responses varied meaningfully across geographic region. Fig. A displays a map of these respondent locations. The size of points corresponding to geolocation is proportional to the logged number of respondents at each location. Of our 3,782 respondents, 2,012 (53%) were located within the United States, 1,578 (42%) were located in India, and the remainder spanned across different regions including North and South America, Europe, Africa, and Asia.

Location coordinates were obtained using open source software to generate longitude and latitude coordinates from IP address information. For additional information about the geolocation service, see `https://freegeoip.net/?q=130.203.101.234`. The figure was created using the ggplot2 [4] and maps [5] packages in R, and uses geographic data from the CIA World Data Bank II. Note that not all IP addresses can be precisely located (particularly within the developing world) and that it is possible for respondents to mask their true location using false IP addresses. IP addresses that could not be precisely located within a country were placed at the geographic center of that country. Nevertheless, the information we obtained should be sufficient to provide a basic understanding of where crowdsourced workers reside.

Each of our expert coders originated from the United States, so it is possible that the classifications they provided were the product of national or regional biases. It is equally possible that the quality of responses obtained from crowdsourced workers varies by location as a result of factors such as language barriers and the fact that the money paid to complete the task is valued differently across economies. While it is outside the bounds of this study to adjudicate between these potential causes, we do evaluate whether accuracy figures meaningfully vary between US and non-US respondents.

We find that US respondents are, on average, better able to reproduce the expert

Fig. A in S1 File: Geographic Distribution of Respondents

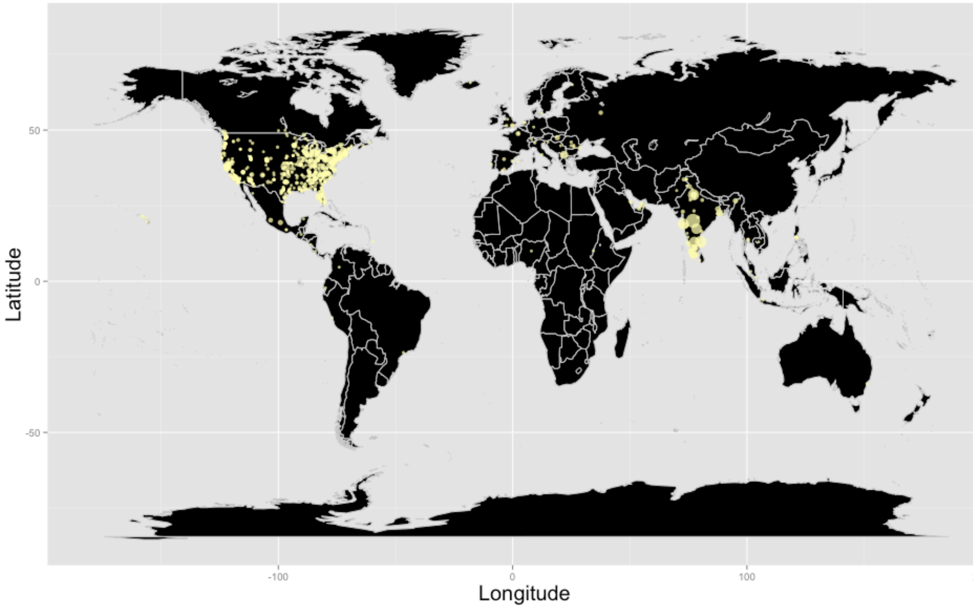

answers when compared to the sample of non-US respondents. Table C reports the accuracy for each of our fields of interest across US and non-US respondents. These figures are reported with respect to both worker-level accuracy and the accuracy obtained when taking a plurality vote (i.e. modal response) among the workers for each story. At the worker level, the responses from US respondents are between 5 and 11 percent more accurate than they are for workers from outside the U.S. The difference between these groups is greatest when workers are asked to identify which state was the initiator of a militarized action, and what precisely that action was. When calculating overall accuracy, or the ability to correctly identify the initiator, target, and militarized action taking place, US respondents are 8 percentage points higher than non-US respondents.

When using voting to aggregate worker responses, the disparity between US and non-US respondents widens or shrinks greatly, depending on the generality of the information being collected. For example, when simply asking determining whether the story contained information about any kind of militarized incident, the difference in accuracy between these two groups is only three percentage points apart. Similarly, this difference is only four percentage points when seeking to identify the hostility level of the events described in the news story (recall that hostility level is a four point index

that identifies whether a story was a non-event, or contains information about a threat, display, or use of force). For more specific fields, however, the gulf widens considerably. The modal response for US workers identifying the initiator and target are 14 and 17 percentage points higher, respectively. Similarly, the modal response pertaining to the specific type of militarized action described in the story is 12 percentage points higher among US respondents. Finally, the overall accuracy is sixteen percentage points higher for US respondents compared to non-US respondents.

Table C in S1 File: Response Accuracy Among U.S. and Non-U.S. Workers

	**Worker**		**Voting**	
	Non-U.S.	U.S.	Non-U.S.	U.S.
Initiator	0.53	0.63	0.58	0.72
Target	0.54	0.61	0.56	0.73
Both	0.50	0.57	0.54	0.72
Incident	0.65	0.70	0.72	0.75
Hostility Level	0.56	0.63	0.67	0.71
Action Type	0.46	0.57	0.56	0.68
Overall	0.41	0.49	0.50	0.66

These results clearly indicate that responses from US workers tend to be more accurate than those collected elsewhere (predominantly India). As previously stated, there are a myriad of factors that might explain the heterogeneity of these two groups, and we leave it to future research to probe this issue further. Nevertheless, our findings along this dimension suggest that practitioners of crowdsourcing methods should be cognizant of where workers are being drawn from. While non-US respondents produced reasonably accurate results, they lacked some precision when identifying fields pertaining to specific actors or action types. As demonstrated in the main text, however, we find that even this relatively noisy information can be used effectively when conducting complex measurement tasks.

3.3 Crowdsourcing and Resource Allocation

For many conflict data projects, resource allocation is a major concern. It is not efficient for trained, expert research assistants to spend their time coding simple cases. It is more efficient for experts to spend their time on the more difficult edge cases and leave the simpler ones to non-experts. However, it is difficult to know *ex ante* which cases will be the most challenging for coders.

Crowdsourcing provides a built-in feature for predicting and separating the easy cases from the more difficult ones–a variable we call *agreement*. *Agreement* measures the unanimity of the responses for each document. *Agreement* can be thought of as a measure of dispersion in the uniformity of crowd responses. For simplicity, we might operationalize *agreement* as the difference in the number of votes received by the leading category and the category that received the second most votes. For example, if three workers labeled a news story as an *attack*, and four as a *clash*, then *agreement* equals one. We find that as the crowd's overall coding decision becomes increasingly unanimous, the crowd's accuracy rapidly increases.

Fig. B in S1 File: Crowd Agreement and Coding Accuracy

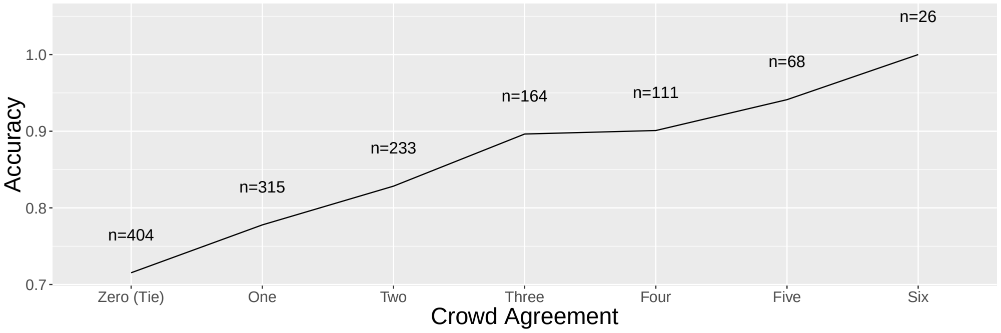

Fig. B plots the accuracy of the crowd against *agreement*. The first point represents the accuracy after discarding all documents for which *agreement* equals zero (i.e., a voting tie). After discarding these documents, n drops from 446 to 404. The remaining points represent the accuracy as *agreement* increases towards six. Notice that as voter unanimity increases, so does the accuracy. At the final point, where just twenty-six documents are not discarded, all twenty-six have been annotated correctly.

Fig. C shows accuracy plotted as a function of *agreement*, when *agreement* is measured using the Herfindahl Index, as shown in Equation 1:

$$Herf = \sum_{i=1}^{n} \left(\frac{freq_i}{responses}\right)^2 \tag{1}$$

The Herfindahl index is used here to measure the concentration of the crowd's responses. In Equation 1, i corresponds to a category of response, n is the number of categories of responses, $freq_i$ is the number of workers who answered category i, and *responses* is the total number of crowd responses. If every response was the same, the

Herfindahl index would be at its maximum of 1. 235

Fig. C in S1 File: Crowd Agreement: Herfindahl Index

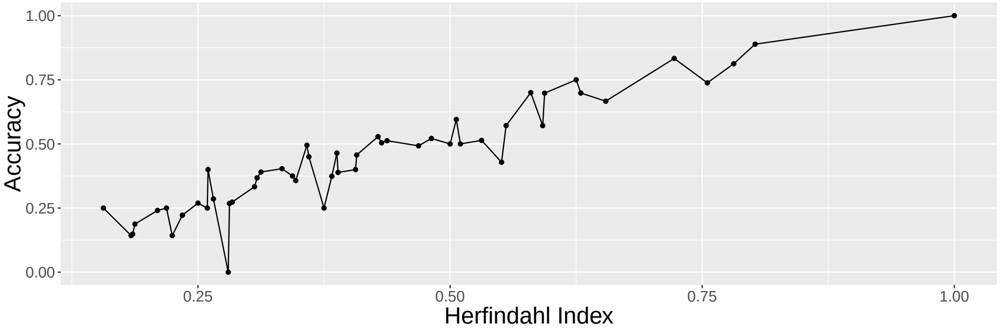

Thus, *agreement* can be used to predict which documents the crowd has coded correctly, and which may have been more difficult or confusing to coders. Documents whose *agreement* is low could be sent to an expert reviewer for further coding, while the crowd's coding might be acceptable for documents whose level of *agreement* is high. Of course, the strength of the relationship in Fig. C will vary depending on the project. Nonetheless, as data collection efforts in the field of conflict studies incorporate technological advances to improve efficiency, as well as to improve the quality and quantity of data, variables such as *agreement* could prove valuable in deciding where and how to allocate the project's finite resources.

4 Crowdsourcing Questionnaire

Below is the full questionnaire hosted on Qualtrics. Note that while the questionnaire is presented here in its entirety, the interactive questionnaire is dynamic, such that individual workers only complete certain portions of the questionnaire, based on their individual responses. As such, while the questionnaire here provides all options potentially available to respondents, workers only see the necessary and relevant components at any given time.

AMT ID Check

Please copy your Amazon Mechanic Turk ID in the box provided. If you have not copied it down from the previous screen, you may locate your AMT ID through Amazon's Mechanical Turk.

◯ Enter your AMT ID below.

Test Block

Your AMT ID is recorded as: ${e://Field/AMTID}

Our records indicates that this is the first time you are taking our survey. Please click the following link, read the story, and answer a few sample questions about it.

MID Story Sample 2

Which of the following best characterizes the action(s) described in the story?

◯ Use of Force
- - Violent use of military forces (armies, warships, planes, etc) by one country against another country
- - Intentional damage to the territory, military, citizens, or goods of another country

◯ Display of Force
- - A non-violent action taken by the military of one country as a show of strength against another country

◯ Threat to Use Force
- - Verbal statements by one country declaring that an action by another country will result in violent or damaging consequences.

◯ None of the Above

In the sample story, did the military forces of one country fire upon or cause damage to the military, population, or territory of another country?

◯ Yes

◯ No

Did the regular military forces of the two countries exchange fire with each other?

◯ Yes

◯ No

[redacted]

The following two questions are general knowledge questions and are not related to the story you have just read.

253

Which of the following is NOT a country?

- ◯ Pakistan
- ◯ India
- ◯ United Nations

Which of the following is NOT considered official military forces?

- ◯ air force
- ◯ navy
- ◯ local militia

First Set

If you're here, then you've either answered our test questions correctly or you have already answered them correctly in a previous survey. Congratulations! You will be directed to a new story by copying the link below. Thank you for participating!

When taking our survey, please disregard any outside knowledge you may have about the event being discussed. We simply want you to read the story and answer questions based solely on the information provided in the document.

We only want you to answer questions about the most recent events described in the story. For example, if the document states that Country A bombed Country B today and this is the first bombing since 10 days ago, your responses should reflect today's bombing only.

Remember, you'll receive a 25 cent bonus for complete and correct answers to our survey!

Thank you!

Please read the following story and answer the remaining questions based only on information contained in this story.

NOTE: Open the link in a new tab/window. You may have to try loading the page more than once.

${e://Field/channel.item.link}

The following questions are being asked to collect information on military actions taken by one country directed against the military, population, or territory of another country.

While these incidents may involve non-state actors such as armed rebel groups or terrorist organizations as a target, we are not directly concerned with the actions these groups take.

254

The following questions, therefore, pertain to conflicts between political and/or military personnel of two countries.

What country or countries (or their military personnel) initiated the action? If civilians or non-state actors (NSA) initiated an action, focus instead on the action a country took in response.

A non-state actor is an entity that is not an official representative of any country. Examples of non-state actors include Al Qaeda, Tamil Tigers, and the Red Cross. Insurgents, rebel groups, and terrorist organizations are all NSAs, as are individuals acting independent of a country, religious and humanitarian organizations.

- ☐ ${e://Field/channel.item.mid:meta_entity_0}
- ☐ ${e://Field/channel.item.mid:meta_entity_1}
- ☐ ${e://Field/channel.item.mid:meta_entity_2}
- ☐ ${e://Field/channel.item.mid:meta_entity_3}
- ☐ Other, please enter the name of the initiating country:

Who is the target of the action by the country/countries selected above? The target may be a country, its civilians, its territory, or a non-state actor (NSA) operating within the country.

Either way, please name the country that the action has been directed against.

- ○ ${e://Field/channel.item.mid:meta_entity_0}
- ○ ${e://Field/channel.item.mid:meta_entity_1}
- ○ ${e://Field/channel.item.mid:meta_entity_2}
- ○ ${e://Field/channel.item.mid:meta_entity_3}
- ○ Other, please enter the name of the target country:

What is the specific geographic location of the incident?

In what country did the incident take place?

- ○ ${e://Field/channel.item.mid:meta_entity_0}
- ○ ${e://Field/channel.item.mid:meta_entity_1}
- ○ ${e://Field/channel.item.mid:meta_entity_2}

- ${e://Field/channel.item.mid:meta_entity_3}
- Location is unclear
- Other, please enter the name of the country where the action occurred:

255

In 2-3 sentences, please summarize the actions taken by ${q://QID20/ChoiceGroup/SelectedChoicesTextEntry} targeting ${q://QID5/ChoiceGroup/SelectedChoicesTextEntry}.

Please focus on the most recent or most important events that took place involving the military. Do not go into detail.

Are the events involving the military action best described as physical, material actions (such as an attack by military forces) or verbal statements and comments (such as a meeting among military officials)?

- Physical, material actions
- Verbal statements and comments

Are the events involving the military best described as conflictual and confrontational, or friendly and cooperative?

- Conflictual and confrontational
- Friendly and cooperative

Which of the following best characterizes the military action(s) described in the story? <u>If more than one option applies, select the first of the options listed below.</u>

- **Physical, violent use of military force** [Sample use of force stories]
  - Active (possibly violent) use of military forces (armies, warships, troops, planes, etc.) by one country against another country
  - Intentional damage or harm to the territory, military, citizens, or goods of another county
  - Intentional capture of the territory, military, citizens, or goods of another country
  - Land or border violation of a country's territory by the military of another country
- **Show or display of military force** [Sample display of force stories]
  - A non-violent action taken by the military of a country as a deliberate show of strength against another country
  - Physical violation of another country's national waters or airspace by military forces
  - A country fortifying its border region or sending additional troops to its border
  - A country putting its military forces on alert or a heightened state of readiness
  - A country activating or mobilizing reserve or irregular military forces

Verbal threat to use military force [Sample threat to use force story]

- Verbal statements by one country declaring that an action or actions by another country will result in violent or damaging consequences

None of the above

256

Use of Force Block round4

You've defined ${q://QID20/ChoiceGroup/SelectedChoicesTextEntry} as the initiator of military action and ${q://QID5/ChoiceGroup/SelectedChoicesTextEntry} as the target.

We're now going to ask some questions pertaining to uses of military force.

Did the armed forces of one country fire upon or cause damage to the military, population, or territory of another country?

Yes

No

Did the attacking country use chemical, biological, or radiological weapons? If unsure, or if the story does not state, answer "no."

Yes

No

This story is about the tactical use of chemical, biological, or radiological weapons by ${q://QID20/ChoiceGroup/SelectedChoicesTextEntry} against the territory or forces of ${q://QID5/ChoiceGroup/SelectedChoicesTextEntry} . Is this an accurate depiction of the event?

Yes

No

Yes, but the actors are reversed

This sounds like a conventional attack. Did the military of the target country return fire in any capacity? If unsure, or if the story does not state, answer "no." If a non-state actor returned fire, answer "no."

A non-state actor is an entity that is not an official representative of any country. Examples of non-state actors include Al Qaeda, Tamil Tigers, and the Red Cross. Insurgents, rebel groups, and terrorist organizations are all NSAs, as are individuals acting independent of a country, religious and humanitarian organizations.

Yes

No

This story is about a clash between ${q://QID20/ChoiceGroup/SelectedChoicesTextEntry} and ${q://QID5/ChoiceGroup/SelectedChoicesTextEntry}. A clash is defined as the outbreak of sustained military hostilities or an exchange of fire between the regular armed forces of two or more countries. Is this an accurate depiction of the event?

- Yes
- No
- Yes, but the actors are reversed

You have indicated that ${q://QID20/ChoiceGroup/SelectedChoicesTextEntry} attacked ${q://QID5/ChoiceGroup/SelectedChoicesTextEntry}. Were civilians the target of this attack? If so, was this action protested or responded to militarily by ${q://QID5/ChoiceGroup/SelectedChoicesTextEntry}?

- Civilians were not the target of the attack. **Note: if the territory or military personnel were targeted in the attack choose this option.**
- Civilians were the target of the attack and ${q://QID5/ChoiceGroup/SelectedChoicesTextEntry} **did not** protest or respond militarily in any capacity.
- Civilians were the target of the attack and ${q://QID5/ChoiceGroup/SelectedChoicesTextEntry} **did** protest and/or respond militarily.

You have indicated that ${q://QID20/ChoiceGroup/SelectedChoicesTextEntry} attacked ${q://QID5/ChoiceGroup/SelectedChoicesText Was there any evidence that ${q://QID5/ChoiceGroup/SelectedChoicesTextEntry} condoned the attack or that ${q://QID20/ChoiceGroup/SelectedChoicesTextEntry} and ${q://QID5/ChoiceGroup/SelectedChoicesTextEntr cooperating militarily?

- Yes, ${q://QID5/ChoiceGroup/SelectedChoicesTextEntry} condoned the attack and/or the two countries were cooperating militarily
- No, the attack was not condoned by ${q://QID5/ChoiceGroup/SelectedChoicesTextEntry} and there was no evidence of military cooperation.

You have indicated that the military forces of ${q://QID20/ChoiceGroup/SelectedChoicesTextEntry} attacked ${q://QID5/ChoiceGroup/SelectedChoicesTextEntry}. An attack is defined as the use of regular armed forces of a country to fire upon the armed forces, population, or territory of another country. Is this an accurate depiction of the event?

- Yes
- No
- Yes, but the actors are reversed

Did the armed forces or official personnel of one country capture or seize the goods, citizens, or property of another country? If yes, which of the following best characterizes what is being seized?

Official Material Goods (weapons, military equipment, or government financial assets, papers, or documents)

- Official Personnel (government officials or military personnel)
- Unofficial Goods/Personnel (a country's citizens or the property of those citizens)
- No seizure took place

258

Did the target country respond militarily or say anything to protest the seizure of its unofficial goods? If unsure, or if the story does not state, answer "no."

- Yes
- No

Were the official goods or personnel held by the seizing country for a period of at least 24 hours? If the story does not explicitly state the length of the seizure, use your best judgment.

- Yes
- No

You have indicated that ${q://QID20/ChoiceGroup/SelectedChoicesTextEntry} captured ${q://QID5/ChoiceGroup/SelectedChoicesTextEntry}'s official goods or personnel and held them for a period of 24 hours. Is this an accurate depiction of the event?

- Yes
- No
- Yes, but the actors are reversed

Were the unofficial goods or personnel held by the target country for a period of at least 24 hours? If the story does not explicitly state the length of the seizure, use your best judgment.

- Yes
- No

You have indicated that ${q://QID20/ChoiceGroup/SelectedChoicesTextEntry} captured ${q://QID5/ChoiceGroup/SelectedChoicesTextEntry}'s unofficial goods or personnel, ${q://QID5/ChoiceGroup/SelectedChoicesTextEntry} protested the seizure, and the unofficial goods or personnel were held for a period of at least 24 hours. Is this an accurate depiction of the event?

- Yes
- No
- Yes, but the actors are reversed

Did the military of one country violate the airspace, land, or sea border of another country? If yes, select whether the violation was by air, land, or sea. Otherwise, select no.

- Yes, by air
- Yes, by land
- Yes, by sea
- No violation occurred

259

Were the military forces trespassing for a period longer than 24 hours? If the story does not explicitly state the length of the violation, use your best judgment.

- Yes
- No

You have indicated that ${q://QID20/ChoiceGroup/SelectedChoicesTextEntry} has occupied ${q://QID5/ChoiceGroup/SelectedChoicesTextEntry}. An occupation is defined as the use of military force by one country to occupy all or part of another country's territory for a period of at least twenty-four hours. Is this an accurate depiction of the event?

- Yes
- No
- Yes, but the actors are reversed

You have indicated that ${q://QID20/ChoiceGroup/SelectedChoicesTextEntry} has violated the border of ${q://QID5/ChoiceGroup/SelectedChoicesTextEntry}. A border violation is defined as the crossing of a border by military forces of one country or the occupation of all or part of another country's territory for a period of less than twenty-four hours. Is this an accurate depiction of the event?

- Yes
- No
- Yes, but the actors are reversed

You have indicated that ${q://QID20/ChoiceGroup/SelectedChoicesTextEntry} has violated the border of ${q://QID5/ChoiceGroup/SelectedChoicesTextEntry} by sea or by air. Such a border violation is non-violent but is meant to be threatening or as a show of strength. Is this an accurate depiction of the event?

- Yes
- No
- Yes, but the actors are reversed

Did the military forces of one country blockade another country? A blockade is an attempt to inhibit or impinge upon the flow of goods or personnel into our out of a country?

- Yes
- No

260

The military of ${q://QID20/ChoiceGroup/SelectedChoicesTextEntry} set up a blockade against ${q://QID5/ChoiceGroup/SelectedChoicesTextEntry}. A blockade is defined as the use of military forces by one country to partially or completely seal off the territory of another country to prevent the entry or exit of goods or personnel. Stopping or inspecting ships or vehicles or the confiscation of goods in transit is evidence of the erection of a blockade. A formal declaration is not required. Is this an accurate depiction of the event?

- Yes
- No
- Yes, but the actors are reversed

Did one country explicitly declare or join a war against another country? To join a war, the target country must already be at war with another country.

- Yes, declared war
- Yes, joined a war
- No

${q://QID20/ChoiceGroup/SelectedChoicesTextEntry} has joined an ongoing war against ${q://QID5/ChoiceGroup/SelectedChoicesTextEntry}, who had already been at war with at least one other country. Is this an accurate depiction of the event?

- Yes
- No
- Yes, but the actors are reversed

${q://QID20/ChoiceGroup/SelectedChoicesTextEntry} has declared war against ${q://QID5/ChoiceGroup/SelectedChoicesTextEntry}. ${q://QID5/ChoiceGroup/SelectedChoicesTextEntry} had not previously been at war with any other country. Is this an accurate depiction of the event?

- Yes
- No
- Yes, but the actors are reversed

Display of Force Block round4

Great, thanks for your work. It looks like there is not a physical use of violent, military force in this story. We are now going to ask a few questions about non-violent displays of military force.

261

Did a country fortify its border region, either by constructing new fortifications or sending additional troops to the border region?

- Yes
- No

${q://QID20/ChoiceGroup/SelectedChoicesTextEntry} has fortified its border with ${q://QID5/ChoiceGroup/SelectedChoicesTextEntry}. A border fortification is defined as an explicit attempt to publicly demonstrate control over a border area through the construction or non-routine reinforcement of military outposts in or near the border area. Is this an accurate description of the event?

- No
- Yes, the country fortifying its border is ${e://Field/channel.item.mid:meta_entity_0}
- Yes, the country fortifying its border is ${e://Field/channel.item.mid:meta_entity_1}
- Yes, the country fortifying its border is ${e://Field/channel.item.mid:meta_entity_2}
- Yes, the country fortifying its border is ${e://Field/channel.item.mid:meta_entity_3}

Did a country place its military on alert? In other words, did it increase the readiness of its regular military (not its military reserves)? If yes, the story will usually mention the words "alert" or "heightened readiness" of military forces.

- Yes
- No

Did this increased readiness mention an increased readiness of nuclear forces? If unsure, or if the story does not state, answer "no."

- Yes
- No

${q://QID20/ChoiceGroup/SelectedChoicesTextEntry} activated a nuclear alert. A nuclear alert is defined as an increased readiness of a state's nuclear armed forces. Is this an accurate description of the event?

- No
- Yes, the country on nuclear alert is ${e://Field/channel.item.mid:meta_entity_0}
- Yes, the country on nuclear alert is ${e://Field/channel.item.mid:meta_entity_1}

- Yes, the country on nuclear alert is ${e://Field/channel.item.mid:meta_entity_2}
- Yes, the country on nuclear alert is ${e://Field/channel.item.mid:meta_entity_3}

262

${q://QID20/ChoiceGroup/SelectedChoicesTextEntry} activated a military alert. An alert is defined as an increased readiness of a state's regular armed forces. Is this an accurate description of the event?

- No
- Yes, the country on alert is ${e://Field/channel.item.mid:meta_entity_0}
- Yes, the country on alert is ${e://Field/channel.item.mid:meta_entity_1}
- Yes, the country on alert is ${e://Field/channel.item.mid:meta_entity_2}
- Yes, the country on alert is ${e://Field/channel.item.mid:meta_entity_3}

Did one country mobilize its military? That is, it activated all or part of its previously inactive or reserve military forces?

- Yes
- No

${q://QID20/ChoiceGroup/SelectedChoicesTextEntry} engaged in a mobilization. A mobilization is defined as the activation by a state of all or part of its previously inactive (reserve) armed forces. Is this an accurate description of the event?

- No
- Yes, the country that mobilized is ${e://Field/channel.item.mid:meta_entity_0}
- Yes, the country that mobilized is ${e://Field/channel.item.mid:meta_entity_1}
- Yes, the country that mobilized is ${e://Field/channel.item.mid:meta_entity_2}
- Yes, the country that mobilized is ${e://Field/channel.item.mid:meta_entity_3}

Did any country use its military to make a public demonstration in order to intimidate others? This might be a non-routine military exercises near a target country's border and/or violating another country's waters or airspace.

- Yes
- No

${q://QID20/ChoiceGroup/SelectedChoicesTextEntry} engaged in a show of force against ${q://QID5/ChoiceGroup/SelectedChoicesTextEntry}. A show of force is defined as a public demonstration by a state of its military forces intended to intimidate another state but not involving actual combat. Examples include non-routine military maneuvers and military exercises, naval patrols immediately outside the territorial waters of another state, and then intentional violation of

another state's territorial waters or air space. Is this an accurate description of the event?

- Yes
- No
- Yes, but the actors are reversed

263

Threat of Force Block round 4

Focus only on verbal statements and disregard all physical actions. Did one country verbally threaten another?

A threat is an explicit statement where a ranking official clearly states that his/her country will take physical action if another state engages in a specified type of behavior.

An example of threat would be U.S. President Bush stating, "If Saddam Hussein does not allow weapons inspectors into Iraq, then the U.S. will attack."

An example of a statement that IS NOT a threat would be U.S. President Bush stating "Saddam Hussein must allow weapons inspectors into Iraq.

- Yes
- No

What did the country threaten to do? Select the best choice.

- Use military force
- Blockade
- Occupy territory
- Declare war
- Use chemical, biological, or radiological weapons
- Join a war against the target
- Actually, there is no threat!

${q://QID20/ChoiceGroup/SelectedChoicesTextEntry} has threatened ${q://QID5/ChoiceGroup/SelectedChoicesTextEntry}. Is this an accurate depiction of the event?

- Yes
- No
- Yes, but the actors are reversed

Random Test Question Block

Which of the following is not a country's regular military?

- Air force
- Rebel group
- Coast guard
- Navy

Which of the following would you describe as a ranking government official?

- General
- Businesswoman
- Priest
- Soldier

Which of the following is an internationally recognized country?

- Gaza
- Kashmir
- Sweden
- Puerto Rico

Who is the president of the United States?

- Barry Sanders
- Barack Obama
- Genghis Khan
- Hillary Clinton

Which of the following is not a religion?

- Islam
- Hinduism
- Christianity
- Amnesty

Which of the following is an ocean?

- Atlantic

- American
- United
- JetBlue

265

Aux Questions

As described in this particular story, did the actions taken by either ${q://QID20/ChoiceGroup/SelectedChoicesTextEntry} or ${q://QID5/ChoiceGroup/SelectedChoicesTextEntry} result in any fatalities among the military personnel of either country (non-civilian fatalities)?

- Yes
- No
- Unsure

How many non-civilian fatalities did ${q://QID5/ChoiceGroup/SelectedChoicesTextEntry} suffer? Please enter the exact number. If no exact number is provided, please the box that best estimates the number.

- If exact, enter here:
- None
- 1-25
- 26-100
- 101-250
- 251-500
- 501-999
- >999

How many non-civilian fatalities did ${q://QID20/ChoiceGroup/SelectedChoicesTextEntry} suffer? Please enter the exact number. If no exact number is provided, please the box that best estimates the number.

- If exact, enter here:
- None
- 1-25
- 26-100
- 101-250
- 251-500
- 501-999
- >999

This news report was reported on ${e://Field/channel.item.mid:meta_date}, is this the same date on which the militarized incident described occurred?

266

- Yes
- No

On which date did this militarized incident occur?

Year

Month

Day

Did the militarized incident last longer than one day?

- Yes
- No

On which date did this militarized incident end?

Year

Month

Day

According to the news report, did the military or political officials from ${q://QID20/ChoiceGroup/SelectedChoicesTextEntry} issue an apology over the incident? Did they make conciliatory remarks of any kind towards ${q://QID5/ChoiceGroup/SelectedChoicesTextEntry}?

- Yes
- No

Are there any non-state actors involved in the incident? Otherwise, enter "none." Examples include international organizations such as the United Nations or rebel groups and terrorist organizations such as Al-Qaeda. Please separate the names of actors with a semi-colon (e.g. United Nations ; Al-Qaeda)

- Yes
- No

Completion Block

Did you enjoy this HIT? If so, please enter your e-mail address to be notified when we have additional HITs of this type. We will not give away your e-mail address and will only use it to notify you of HITs.

Feedback

This is not required, but we encourage you to provide us with some feedback about this survey. What you liked and did not like, areas of improvement, errors that you encountered, etc.

The following code is to be copied and pasted into Mechanical Turk so that you get credit for completing this HIT. If you are not given a code, please enter your Mechanical Turk ID instead.

Code: ${e://Field/channel.item.title}

Thank you! Our research benefits from having repeat workers, so if you'd like to accept another HIT, here's a link to get you started: LINK TO MORE HITS

Repeat

Thank you for telling us about this story! Please click the following link to continue working on this HIT. Remember, you have two hours to complete this HIT.

When prompted, copy the following AMT ID: ${e://Field/AMTID}.

Link to the next story.

Repeat2

Thank you for telling us about this story! Please click the following link to continue working on this HIT. Remember, you have two hours to complete this HIT.

When prompted, copy the following AMT ID: ${e://Field/AMTID}.

Link to the next story.

Repeat3

Thank you for telling us about this story! Please click the following link to continue working on this HIT. Remember, you have two hours to complete this HIT.

When prompted, copy the following AMT ID: ${e://Field/AMTID}.

268

Link to the next story.

Repeat4

Thank you for telling us about this story! Please click the following link to continue working on this HIT. Remember, you have two hours to complete this HIT.

When prompted, copy the following AMT ID: ${e://Field/AMTID}.

Link to the next story.

Difficult Test Block

Your AMT ID is recorded as: ${e://Field/AMTID}

Our records indicates that this is the first time you are taking our survey. Please click the following link, read the story, and answer a few sample questions about it.

Difficult test story

The following questions are being asked to collect information on threats, displays, and uses of military force by one country, directed against the military personnel and/or civilians of another. While these incidents may involve non-state actors such as armed rebel groups or terrorist organizations, we are not directly concerned with the actions these groups take. The following questions, therefore, pertain to disputes between political and/or military personnel of two countries.

When taking our survey, please disregard any outside knowledge you may have about the event being discussed. We simply want you to read the story and answer questions based solely on the information provided in the document. Furthermore, we only want you to answer questions about the most recent events described in the story. For example, if the document states that Country A bombed Country B today and this is the first bombing since 10 days ago, we want you to answer questions that reflect today's bombing and not the one that happened 10 days ago.

Which of the following best characterizes the action(s) described in the story?

- ◯ Use of Force
  - - Violent use of military forces (armies, warships, planes, etc) by one country against another country
  - - Intentional damage to the territory, military, citizens, or goods of another country

- ○ Display of Force
  - - A non-violent action taken by the military of one country as a show of strength against another country
- ○ Threat to Use Force
  - - Verbal statements by one country declaring that an action by another country will result in violent or damaging consequences.

- ○ None of the Above

What country was the initiator of the military action?

- ○ TUR
- ○ IRQ
- ○ PKK
- ○ PAK

What country or countries was the target of the military action?

- ☐ TUR
- ☐ PKK
- ☐ PAK
- ☐ IRQ

Were there any non-state actors, such as terrorist groups or multi-national groups, present during the event?

- ○ Yes
- ○ No

Were any uniformed military personnel killed during the event?

- ○ Yes
- ○ No

First Set -- Bypass

If you're here, then you've either answered our test questions correctly or you have already answered them correctly in a previous survey. Congratulations! You will be directed to a new story by clicking the link below. Thank you for participating!

The following questions are being asked to collect information on threats, displays, and uses of military force by one country, directed against the military personnel and/or civilians of

another. While these incidents may involve non-state actors such as armed rebel groups or terrorist organizations, we are not directly concerned with the actions these groups take. The following questions, therefore, pertain to disputes between political and/or military personnel of two countries.

When taking our survey, please disregard any outside knowledge you may have about the event being discussed. We simply want you to read the story and answer questions based solely on the information provided in the document. Furthermore, we only want you to answer questions about the most recent events described in the story. For example, if the document states that Country A bombed Country B today and this is the first bombing since 10 days ago, we want you to answer questions that reflect today's bombing and not the one that happened 10 days ago.

Thank you!

Please read the following story and answer the remaining questions based on information in this story.

NOTE: Open the link in a new tab/window.

${e://Field/channel.item.link}

Use of Force Block

You have indicated that the military forces of ${q://QID20/ChoiceGroup/SelectedChoicesTextEntry} attacked, raided, or bombed ${q://QID5/ChoiceGroup/SelectedChoicesTextEntry}. This is defined as the use of regular armed forces of a country to fire upon the armed forces, population, or territory of another country. Is this an accurate depiction of the event?

◯ Yes

◯ No

You have indicated that the military forces of ${q://QID20/ChoiceGroup/SelectedChoicesTextEntry} have engaged in a violent clash or battle ${q://QID5/ChoiceGroup/SelectedChoicesTextEntry}. A clash is defined as the outbreak of sustained military hostilities between the regular armed forces of two or more countries. Is this an accurate depiction of the event?

◯ Yes

◯ No

Were chemical, biological, or radiological weapons used? If unsure, answer "No."

◯ Yes

◯ No

You have indicated that ${q://QID20/ChoiceGroup/SelectedChoicesTextEntry} has seized goods or personnel of ${q://QID5/ChoiceGroup/SelectedChoicesTextEntry}. Which of the following best characterizes the type of property that was seized:

- Weapons, military equipment, or government financial assets, papers, or documents
- Government officials or military personnel
- A country's citizens or the property of those citizens

271

Did ${q://QID5/ChoiceGroup/SelectedChoicesTextEntry} protest this seizure? Methods of protest include filing a complaint with ${q://QID20/ChoiceGroup/SelectedChoicesTextEntry} or an international organization, making public statements indicting ${q://QID20/ChoiceGroup/SelectedChoicesTextEntry}, or taking military action against ${q://QID20/ChoiceGroup/SelectedChoicesTextEntry} in response to their seizure.

- Yes
- No

Were the seized goods or personnel captured and held by ${q://QID20/ChoiceGroup/SelectedChoicesTextEntry} for a period of at least 24 hours?

- Yes
- No

Were the seized goods or personnel captured and held by ${q://QID20/ChoiceGroup/SelectedChoicesTextEntry} for a period of at least 24 hours?

- Yes
- No

You have indicated that ${q://QID20/ChoiceGroup/SelectedChoicesTextEntry} captured or seized ${q://QID5/ChoiceGroup/SelectedChoicesTextEntry}'s goods or personnel and held them for a period of 24 hours. Is this an accurate depiction of the event?

- Yes
- No

You have indicated that ${q://QID20/ChoiceGroup/SelectedChoicesTextEntry} has blockaded ${q://QID5/ChoiceGroup/SelectedChoicesTextEntry}. A blockade is defined as the use of military forces by one country to partially or completely seal off the territory of another country to prevent the entry or exit of goods or personnel. Is this an accurate depiction of the event?

- Yes
- No

You have indicated that the military forces of ${q://QID20/ChoiceGroup/SelectedChoicesTextEntry} illegally crossed the border of ${q://QID5/ChoiceGroup/SelectedChoicesTextEntry}, either as a border violation or as an occupation of territory. Was this for a period of <u>less than</u> 24 hours?

- ◯ Yes
- ◯ No

This is a border violation. A border violation is defined as the use of military force by one country to occupy all or part of another country's territory for a period of <u>less than</u> twenty-four hours. Is this an accurate depiction of the event?

- ◯ Yes
- ◯ No

This is an occupation of territory. An occupation is defined as the use of military force by one country to occupy all or part of another country's territory for a period of <u>at least</u> twenty-four hours. Is this an accurate depiction of the event?

- ◯ Yes
- ◯ No

Display of Force Block

You have indicated that ${q://QID20/ChoiceGroup/SelectedChoicesTextEntry} has fortified its border with ${q://QID5/ChoiceGroup/SelectedChoicesTextEntry}. A border fortification is defined as an explicit attempt to publicly demonstrate control over a border area through the construction or non-routine reinforcement of military outposts in or near the border area. Is this an accurate description of the event?

- ◯ Yes
- ◯ No

You have indicated that ${q://QID20/ChoiceGroup/SelectedChoicesTextEntry} has mobilized its troops or placed them on alert with ${q://QID5/ChoiceGroup/SelectedChoicesTextEntry} as the target. As part of this mobilization or alert, were previously inactive or reserve troops activated by ${q://QID20/ChoiceGroup/SelectedChoicesTextEntry}?

- ◯ Yes
- ◯ No

You have identified a mobilization by ${q://QID20/ChoiceGroup/SelectedChoicesTextEntry}

against ${q://QID5/ChoiceGroup/SelectedChoicesTextEntry}. A mobilization is defined as the activation by a country of all or part of its previously inactive or reserve forces. Is this an accurate depiction of the event?

- Yes
- No

You have identified an alert by ${q://QID20/ChoiceGroup/SelectedChoicesTextEntry} against ${q://QID5/ChoiceGroup/SelectedChoicesTextEntry}. An alert is defined as an increase in the military readiness of a country's regular armed forces. Is this an accurate depiction of the event?

- Yes
- No

A <u>nuclear alert</u> is defined as an increased readiness of a country's <u>nuclear</u> armed forces. Is this an accurate description of the event?

- Yes
- No

You have indicated that ${q://QID20/ChoiceGroup/SelectedChoicesTextEntry} has made a public demonstration intended to intimidate ${q://QID5/ChoiceGroup/SelectedChoicesTextEntry}, but not involving physical combat. This includes border violations by air or sea, naval patrols immediately outside the territorial waters of ${q://QID5/ChoiceGroup/SelectedChoicesTextEntry}, and non-routine or controversial military maneuvers or exercises. Is this an accurate depiction of the event?

- Yes
- No

Did ${q://QID5/ChoiceGroup/SelectedChoicesTextEntry} respond with a public demonstration intended to intimidate ${q://QID20/ChoiceGroup/SelectedChoicesTextEntry}? In other words, are ${q://QID5/ChoiceGroup/SelectedChoicesTextEntry} and ${q://QID20/ChoiceGroup/SelectedChoicesTextEntry} engaging in mutual standoff-ish behavior?

- Yes
- No

Threat of Force Block

You have indicated that ${q://QID20/ChoiceGroup/SelectedChoicesTextEntry} has threatened ${q://QID5/ChoiceGroup/SelectedChoicesTextEntry}. A threat is an explicit statement where a ranking official clearly states that his/her country will take violent, physical action if another state engages in a specified type of behavior.

An example of a threat would be U.S. President Bush stating "If Saddam Hussein does not allow weapons inspectors into Iraq, the U.S. will attack."

An example of a statement that is not a threat would be U.S. President Bush stating "Saddam Hussein must allow weapons inspectors into Iraq."

274

In light of this definition of a threat, is it accurate to say that ${q://QID20/ChoiceGroup/SelectedChoicesTextEntry} has threatened ${q://QID5/ChoiceGroup/SelectedChoicesTextEntry}?

- Yes
- No

You have indicated that ${q://QID20/ChoiceGroup/SelectedChoicesTextEntry} has threatened to use military force against ${q://QID5/ChoiceGroup/SelectedChoicesTextEntry}. This is defined as the threat by one country to use its regular armed forces to fire upon the armed forces or violate the territory of another country. Is this an accurate depiction of the event?

- Yes
- No

You have indicated that ${q://QID20/ChoiceGroup/SelectedChoicesTextEntry} has threatened to declare war against ${q://QID5/ChoiceGroup/SelectedChoicesTextEntry}. Is ${q://QID5/ChoiceGroup/SelectedChoicesTextEntry} currently at war with a country other than ${q://QID20/ChoiceGroup/SelectedChoicesTextEntry}?

- Yes
- No

You have indicated that ${q://QID20/ChoiceGroup/SelectedChoicesTextEntry} has threatened to join an ongoing war against ${q://QID5/ChoiceGroup/SelectedChoicesTextEntry}. In other words, ${q://QID5/ChoiceGroup/SelectedChoicesTextEntry} is at war with another country and ${q://QID20/ChoiceGroup/SelectedChoicesTextEntry} has threatened to join that other country against ${q://QID5/ChoiceGroup/SelectedChoicesTextEntry}. Is this an accurate depiction of the event?

- Yes
- No

You have indicated that ${q://QID20/ChoiceGroup/SelectedChoicesTextEntry} has threatened to declare war against ${q://QID5/ChoiceGroup/SelectedChoicesTextEntry}, who is not at war with any other country. Is this an accurate depiction of the event?

- Yes
- No

Did ${q://QID20/ChoiceGroup/SelectedChoicesTextEntry} verbally threaten to use chemical, biological, or radiological (nuclear) weapons against ${q://QID5/ChoiceGroup/SelectedChoicesTextEntry}?

275

- Yes
- No

You have indicated that ${q://QID20/ChoiceGroup/SelectedChoicesTextEntry} has threatened to occupy the territory of ${q://QID5/ChoiceGroup/SelectedChoicesTextEntry}. An occupation of territory is defined as the use of military force to occupy all or part of another country's territory for at least 24 hours. Is this an accurate depiction of the event?

- Yes
- No

You have indicated that ${q://QID20/ChoiceGroup/SelectedChoicesTextEntry} has threatened to blockade ${q://QID5/ChoiceGroup/SelectedChoicesTextEntry}. A blockade is defined as the use of military forces to partially or completely seal off the territory of another country to prevent the entry or exit of goods or personnel. Is this an accurate depiction of the event?

- Yes
- No

You have indicated that ${q://QID20/ChoiceGroup/SelectedChoicesTextEntry} has threatened ${q://QID5/ChoiceGroup/SelectedChoicesTextEntry} in some way that does not fit into our schema. Please describe the threat and be specific.

Aux Questions -- Bypass

The attached story does not detail any threats, displays or uses of military force between two or more countries. Which of the following, then, best characterize the context of the story?

- A country is engaged in routine military operations
- Two or more countries are holding joint military operations
- One or several countries are engaged in combat operations against non-state entities (i.e. rebel groups or terrorist organizations)
- A sporting event is taking place
- None of the above

This news report was reported on ${e://Field/channel.item.mid:meta_date}, is this the same date on which the militarized incident described occurred?

- Yes
- No

276

On which date did this militarized incident occur?

Year
Month
Day

Did the militarized incident last longer than one day?

- Yes
- No

On which date did this militarized incident end?

Year
Month
Day

Was the military action described in the story taken to resolve an underlying issue between the countries involved? If so, mark which category best describes the issue below.

- **Territory**
  - \- A country is attempting to gain control of a land-based piece of territory that it does not currently possess.
- **Regime**
  - -A country is attempting to change or overthrow the government of another country.
- **Policy** - A country is attempting to change the behavior of another country with respect to a specific policy. This includes issues surrounding maritime and airspace borders. For example a country might use military force to :
  - \- Prohibit citizens from another country from engaging in economic activities on its land or waters.
  - \- End terrorist/rebel activity taking place in another (usually neighboring) country without the expressed concent of that country.
  - \- Prevent another country from entering airspace it claims as its own.
- Other/None

Is there a secondary issue-type that is also underlying this dispute? If so, please select the

secondary issue type below.

- ◯ Territory
  - - A country is attempting to gain control of a land-based piece of territory that it does not currently possess.
- ◯ Regime
  - -A country is attempting to change or overthrow the government of another country.
- ◯ Policy
  - -A country is attempting to change the behavior of another country with respect to a specific policy. This includes issues surrounding maritime and airspace borders.
- ◯ No secondary issue-type

According to the news report, did the military or political officials from ${q://QID20/ChoiceGroup/SelectedChoicesTextEntry} issue an apology over the incident?

- ◯ Yes
- ◯ No

What is the geographic location of the incident? Be as specific as possible.

If there are any non-state actors that are directly involved in the incident detailed in the news report, please enter their name below. Otherwise, enter "none." Examples include international organizations such as the United Nations or rebel groups and terrorist organizations such as Al-Qaeda. Please separate the names of actors with a semi-colon (e.g. United Nations ; Al-Qaeda)

It has been indicated that ${q://QID20/ChoiceGroup/SelectedChoicesTextEntry} has used military force against ${q://QID5/ChoiceGroup/SelectedChoicesTextEntry}. Did ${q://QID5/ChoiceGroup/SelectedChoicesTextEntry} retaliate? In other words, based on your reading of the story, did ${q://QID5/ChoiceGroup/SelectedChoicesTextEntry} respond to ${q://QID20/ChoiceGroup/SelectedChoicesTextEntry} with the use, display, or threat of military force? Please describe ${q://QID5/ChoiceGroup/SelectedChoicesTextEntry}'s response.

It has been indicated that ${q://QID50/ChoiceGroup/SelectedChoicesTextEntry} has displayed military force against ${q://QID52/ChoiceGroup/SelectedChoicesTextEntry}. Did ${q://QID52/ChoiceGroup/SelectedChoicesTextEntry} retaliate? In other words, based on your reading of the story, did ${q://QID52/ChoiceGroup/SelectedChoicesTextEntry} respond to ${q://QID50/ChoiceGroup/SelectedChoicesTextEntry} with the use, display, or threat of military

force? Please describe ${q://QID52/ChoiceGroup/SelectedChoicesTextEntry}'s response.

278

You have indicated that ${q://QID40/ChoiceGroup/SelectedChoicesTextEntry} has threatened to use military force against ${q://QID41/ChoiceGroup/SelectedChoicesTextEntry}. Did ${q://QID41/ChoiceGroup/SelectedChoicesTextEntry} retaliate? In other words, based on your reading of the story, did ${q://QID41/ChoiceGroup/SelectedChoicesTextEntry} respond to ${q://QID40/ChoiceGroup/SelectedChoicesTextEntry} with the use, display, or threat of military force? Please describe ${q://QID41/ChoiceGroup/SelectedChoicesTextEntry}'s response.

In 2-3 sentences, please describe the incident you have just read about.

References

1. Schrodt PA. TABARI: Textual Analysis By Augmented Replacement Instructions; 2014. http://goo.gl/72XFle.

2. Schrodt PA. Conflict and Mediation Event Observations (CAMEO) Codebook; 2012. http://goo.gl/xftKkl.

3. Georgia accuses Russia of violating its air space. Russia & CIS General Newswire. March 5, 2009; LexisNexis Academic. Web. Date Accessed: 2016/01/06.

4. Wickham H. ggplot2: elegant graphics for data analysis. Springer New York; 2009. Available from: http://had.co.nz/ggplot2/book.

5. Becker RA, Wilks AR, Brownrigg R, Minka TP. maps: Draw Geographical Maps; 2014. R package version 2.3-9. Available from: http://CRAN.R-project.org/package=maps.
